# Engineering Wheat for Compound Climate Extremes: Molecular Trade-Offs, Stress Memory, and Precision Genome Editing for Yield-Stable Resilience

**DOI:** 10.3390/plants15192936

**Published:** 2026-09-25

**Authors:** Xin Song, Xixiong Hong, Huaiyuan Wu, Peipei Su, Minshan Sun

**Affiliations:** 1 School of Life Science and Environmental Resources, Yichun University, Yichun 336000, China; 2 College of Plant Protection, Henan Agricultural University, Zhengzhou 450002, China

**Keywords:** compound stress, genome editing, homoeologue dosage, stress memory, trade-offs, wheat, yield stability

## Abstract

Bread wheat (*Triticum aestivum* L.) increasingly encounters combinations of abiotic stresses that interact in time and whose effects are not predictable from single-stress responses. We define yield-stable resilience as the capacity to maintain grain yield under compound climate extremes without a significant penalty in yield or quality under favourable conditions; both components are required, and a line that tolerates stress while losing yield potential does not satisfy the definition. This critical review addresses three dimensions: the temporal architecture of stress across development; the biological control layers that run from perception through recovery to stress memory; and the precision-engineering strategies enabled by genome editing. We argue that field-level yield stability is rarely achieved by constitutively overexpressing or deleting a single stress-response gene. Instead, stress-integration networks must be engineered in a context-dependent, dosage-aware manner. To explain why single-gene solutions fall short, we examine molecular and physiological trade-offs, including water conservation versus transpirational cooling, photoprotection versus carbon acquisition, and stay-green versus remobilization. Stress-memory mechanisms, which range from chromatin modifications to persistent signalling states, offer a route to primed protection, but causal validation in wheat remains largely absent. The hexaploid wheat genome affords distinctive engineering opportunities through homoeologue-dosage control, promoter tuning and tissue-specific regulation. We propose an evidence-gated pathway that proceeds from mechanism to calibrated editing, to biologically realistic stress testing, to multi-environment yield testing, and finally to equitable deployment, and we treat breeding integration, introgression, recurrent selection and precision editing as a single pipeline rather than as competing alternatives.

## 1. Introduction

Bread wheat supplies roughly 20% of the dietary protein and calories consumed globally and underpins food security across temperate and subtropical production regions [1]. Climate change is not only raising mean temperatures; it is also reorganising the temporal structure of stress exposure, and terminal drought, heatwaves, waterlogging followed by rapid soil drying, and multiple extremes within a single growing season are all becoming more frequent [2,3]. Compound, sequential and recurrent stresses of this kind generate biological states unlike those produced by any single stress applied in isolation, yet most mechanistic knowledge derives from precisely such single-stress experiments [4,5].

Three developmental windows concentrate wheat’s climate vulnerability. Vegetative-stage stress, although less directly damaging, alters canopy architecture, root structure and reserve pools that later support reproductive capacity [6]. Spike fertility and grain number are determined at meiosis and anthesis, when stress causes irreversible floret abortion and pollen sterility [7,8]. Stress during grain filling reduces kernel weight and quality by inhibiting starch deposition and protein accumulation [9]. Engineering approaches must therefore account both for the sensitivity of each phenological stage and for whole-plant function across the temporal sequence of stresses.

Survival, biomass maintenance and stress-marker expression measured under a single stress in controlled conditions are inadequate measures of engineered resilience [10]. Yield stability, the capacity to produce grain of acceptable quality across variable environments, integrates stress resistance, recovery after stress relief, completion of grain filling and productivity in favourable seasons [11]. Constitutive overexpression of transcription factors or downstream stress-responsive genes, the most widely used strategy, has yielded plants that survive severe stress but yield poorly under both stress and non-stress conditions [12,13]. Single-gene ‘stress-tolerance’ narratives systematically underestimate growth–defence trade-offs, developmental context dependence and homoeologue complexity [14].

Two fields have developed in parallel. Stress-memory research has shown that chromatin modification, persistent signalling states and metabolic priming can alter responses to a second exposure, but engineering applications remain largely theoretical [15,16] (M/R). Meanwhile, precision editing tools for wheat, including CRISPR nucleases, base editors, prime editors and transcriptional modulators, now permit increasingly sophisticated genetic manipulation [17,18]; however, most traits so far reported in edited wheat are simple agronomic or quality traits rather than stress interactions, and the two fields have rarely been brought together [19,20].

We argue here that resilience of wheat yield to compound climate extremes is rarely delivered by activating or knocking out individual stress-responsive genes. It requires engineering that is context-, stress- and recovery-aware, and that simultaneously supports protection, reproduction, recovery, grain filling and performance in the absence of stress. We develop this argument across three dimensions: the temporal stress architecture that defines the biological challenge (Section 3); the trade-offs that constrain stress responses (Section 4 and Section 5); and the precision-engineering strategies through which those responses can be calibrated (Section 6 and Section 7). We then link all three to field-level yield stability (Section 8). Because the allelic variation on which any of these strategies depends must ultimately come from somewhere, we also treat germplasm resources and breeding integration—introgression, recurrent selection and precision editing—as a single pipeline (Section 7.5) [21].

## 2. Scope and Evidence Framework

### 2.1. Search Strategy, Screening and Reporting Standard

This review is a critical narrative synthesis, not a systematic review or meta-analysis; a structured but non-exhaustive search was used for reproducibility, and no effect sizes are pooled. Searches were conducted in June 2026 in Web of Science, Scopus, PubMed and Google Scholar using three concept groups: species (wheat, *Triticum aestivum*, *Triticum durum*, barley, rice, maize); stress context (compound, combined, sequential and recurrent stress, stress memory, priming, acclimation); and intervention and phenotype (genome editing, CRISPR, base and prime editing, homoeologue, yield stability, yield components, grain filling). No start date was imposed, the language was restricted to English, and backward citation tracking supplemented the database searches [22].

Screening proceeded in two rounds. Records unrelated to compound abiotic stress in wheat were excluded on title and abstract. The remainder were read in full against three inclusion criteria: (a) the study organism was wheat or a related cereal capable of providing mechanistic support; (b) the temporal structure of the stress treatment was explicitly described as concurrent, sequential, recurrent or single; and (c) at least one phenotype related to yield, a yield component or recovery was reported. Only records satisfying all three criteria entered evidence classification; the remainder were retained as contextual citations, not as a basis for a claim. The search strategy, screening flow and record numbers at each stage are given in Table S5 [23].

### 2.2. Terminology and Minimum Design Requirements

Terms frequently used interchangeably are distinguished strictly here; definitions and the minimum experimental design required for each are given in Tables S1 and S6 [24].

Compound stress denotes two or more abiotic stresses acting on the same plant according to a defined temporal structure. Stresses are concurrent when they overlap in time, sequential when they are separated in time and the first modifies the effect of the second, and recurrent when the same stress occurs repeatedly and may engage stress memory. Acclimation denotes adjustment of the response baseline under sustained or repeated stress; priming denotes a change in the magnitude or speed of the response to a later exposure caused by an earlier one. Somatic memory is confined to the lifetime of an individual plant, whereas transgenerational inheritance denotes an effect detectable in unexposed generations. Intergenerational effects—the direct influence of parental exposure on the F1 generation, including maternal and seed-provisioning effects—are not equivalent to transgenerational inheritance: much of what the literature describes as transgenerational memory is in fact intergenerational, and where maternal and seed effects have not been excluded the term transgenerational should not be used [25,26,27].

Tolerance denotes the maintenance of function under a given stress, resistance reduction in the damage caused by stress, recovery the rate and extent of functional restoration after stress relief, and resilience the composite outcome of the three [28]. Yield-stable resilience is defined here as the capacity to maintain grain yield under compound climate extremes without a significant penalty in yield or quality under favourable conditions; both components are required, since maintaining yield under stress while losing yield potential under favourable conditions does not satisfy the definition. Gene symbols follow wheat nomenclature, with homoeologue copies distinguished by -A, -B and -D suffixes (for example, TaDREB2-A); the full name is given at first mention and the abbreviation thereafter. Exposure protocols must state whether exposure was concurrent, sequential or recurrent; studies reporting only single-exposure responses are treated as contextual rather than definitive [29,30].

### 2.3. Evidence Hierarchy and Species-of-Origin Markers

A five-level hierarchy of evidence is applied, defined in full in Table S1 and spanning from Level A (edited or engineered wheat validated across multiple field environments) to Level E (mechanistic evidence from a model species not validated in wheat); Levels B to D denote the intermediate grades of wheat and other-cereal evidence, defined individually in Table S1. This hierarchy is our own construction, adapted from crop genome-editing reviews, and is not an established community standard; it helps the reader judge the strength of evidence behind each claim rather than supplying an accepted criterion [31].

Classification alone does not prevent cross-species inference from being presented as wheat evidence. A species-of-origin marker is therefore applied alongside the evidence level: W denotes direct evidence from wheat; C evidence from another cereal such as barley, rice or maize; M evidence from a model species such as *Arabidopsis*; and R a review or meta-analysis. Evidence marked C or M may only generate hypotheses and may not directly support a claim that a mechanism operates or can be engineered in wheat; such sentences must be phrased hypothetically and must carry the marker. A reference marked R is not primary evidence and may not be the sole support for a claim. This convention matters most for the four categories whose evidence derives largely from C and M sources—ABA signalling, chromatin regulation, promoter editing and stress memory [32,33]. Markers are applied selectively—only where the species of origin could otherwise be mistaken for wheat evidence—and their absence does not imply that the supporting evidence is wheat-derived. Table S7 summarises the markers and their inference rules.

### 2.4. Conceptual Layering: From Climate Hazard to Yield Consequence

A four-level conceptual chain defines the boundary of this review: climate hazards, the meteorological events themselves; the environmental stresses these hazards produce at the crop level; compound stress combinations, in which two or more environmental stresses act according to a defined temporal structure; and their physiological and yield consequences at molecular, physiological and agronomic levels [34,35].

The stresses considered here are not equivalent categories. Heat and drought are climate-related; waterlogging may result from extreme rainfall; salinity, although climate-associated in some production systems such as irrigation-induced salinization under drought, is not itself a climate extreme; and nutrient limitation is primarily a matter of soil management rather than a climate extreme [36,37]. Salinity and nutrient limitation are therefore included as stresses that co-occur with climate extremes in the field and share signalling nodes with them, not as climate extremes in their own right—a deliberate and bounded extension of the scope rather than an unacknowledged broadening [38,39,40].

## 3. Compound Climate Extremes Create Distinct Biological States

The sections that follow treat compound stress not as a set of independent treatments but as a causal chain: molecular perception and signal integration; the physiological processes these regulate, including water relations, stomatal behaviour, photosynthesis, osmotic and redox homeostasis, source-sink allocation and reproductive development; the yield components those processes determine; and finally yield and its stability across environments. Each subsection addresses one link in this chain while retaining the whole-plant context. Section 3.1 establishes the temporal architectures that define the hazard, Section 3.2 the non-additive molecular and physiological responses they generate, and Section 3.3 the developmental timing that determines whether a given mechanism is protective or damaging [34,35].

### 3.1. Temporal Architecture of Compound Stress

Wheat’s exposure to climate extremes is defined not only by stress intensity but by the temporal organisation of those extremes [34,35]. We distinguish six stress architectures, each demanding a different biological and engineering solution (Figure 1). Concurrent compound stress, in which stressors overlap in time (heat and drought during grain filling, for example), produces interactive responses in water relations, photosynthesis and reproductive development [41,42,43] (W/M). Sequential compound stress comprises temporally separated stressors in which the first alters the response to the second, as when waterlogging during tillering damages the root system and modifies the impact of a subsequent terminal drought [44,45]. Recurrent compound stress, such as repeated heatwaves during reproductive development, may engage stress-memory mechanisms [46]. Chronic background stress with acute extremes describes terminal heatwaves superimposed on moderate, season-long stress [38]. Stress followed by incomplete recovery describes residual effects that constrain performance under subsequently favourable conditions or during a later stress [47]. Finally, stage-specific stress reflects the fact that an identical dose produces different consequences at meiosis, at anthesis and during grain filling [7,9,48].

This temporal complexity challenges the experimental basis of most stress biology [49]. Typical experiments impose stress abruptly, hold it constant and relieve it rapidly; intensity does not build gradually, diurnal variation is absent and prolonged recovery is not monitored, so the resulting states may not represent responses under agriculturally relevant dynamics [50]. Field studies using rainout shelters, heat tents and managed-stress environments are more realistic but remain uncommon and logistically demanding [51]. Figure 1 organises these architectures against wheat phenology and shows that stresses arising at different stages, or in sequence, carry different molecular, physiological and agronomic consequences [52].

### 3.2. Non-Additive Molecular and Physiological Responses

Because nodes of stress perception and signalling converge, compete or antagonise one another, compound-stress phenotypes cannot be predicted by summing individual stress responses [53,54]. Concurrent heat and drought, the most agriculturally important compound stress in wheat, illustrates this. Under drought, the canonical water-conserving response is abscisic acid (ABA)-mediated stomatal closure, which limits water loss but reduces transpirational cooling and so raises leaf temperature [55]. Some genotypes instead maintain stomatal opening as temperature rises, favouring evaporative cooling [56]. When the two stresses coincide, the drought-induced ABA response can dominate, leaving leaves exposed to higher temperatures, so that the outcome is synergistic rather than additive [57,58]. Consistent with this, transcriptomic studies of combined heat and drought indicate that a substantial fraction of the differentially regulated genes respond uniquely to the combination rather than to either single stress, but the reported proportion varies widely with genotype, tissue, sampling time and stress intensity, spanning approximately one-sixth to more than half of the responsive genes depending on the experimental design [41,54,59] (C/M). Non-additivity is therefore a robust qualitative conclusion, whereas any single percentage should not be treated as a general property of combined heat–drought responses. This variability is itself informative: it shows that the transcriptional outcome of combined stress depends strongly on the experimental design, and it reinforces the argument in Section 3.3 that the stress architecture of an experiment must be reported in a standardised form [49,50].

Waterlogging followed by drought is a second, increasingly common scenario [39,40]. Waterlogging causes root hypoxia, reducing hydraulic conductivity and impairing nutrient uptake [45]; when drought follows, the damaged root system may be unable to meet the transpirational load even where soil moisture remains available, producing greater susceptibility than in drought-naive plants [44,60]. By contrast, moderate drought priming can confer cross-tolerance to subsequent heat through sustained ABA signalling, osmolyte accumulation and elevated antioxidant capacity [46,61].

At the molecular level, non-additivity arises from shared nodes whose function is context-specific [62]. Calcium signatures differ qualitatively between single and combined stress, with stress identity encoded in oscillation frequency and amplitude [63]. Reactive oxygen species (ROS) signalling is central to stress perception and acclimation but becomes damaging when combined stresses outpace antioxidant capacity [64]. Hormones including ABA, jasmonic acid, salicylic acid, ethylene and cytokinins transmit environmental signals and interact antagonistically with one another, constraining adaptation to concurrent stresses [29,65,66]. Energy-status signalling through SnRK1 and TOR kinases governs the allocation of resources between growth and defence, and combined stresses deplete energy reserves more rapidly than single stresses [67].

### 3.3. Developmental Timing and Yield Consequences

Developmental timing is among the principal determinants of the yield impact of stress. When stress intensity and duration are comparable, the stage at which stress occurs generally explains the largest share of the resulting yield loss [9], but this ordering is not universal: intensity, duration, genotype and recovery capacity also shape the yield response, and at extreme intensities the effect of intensity can exceed that of timing. Heat during meiosis causes chromosome mis-segregation and pollen inviability, whereas drought at anthesis impairs pollen tube growth and ovule fertilisation [7,8]; combined heat and drought in this window can cause near-total loss of spike fertility in sensitive varieties [68]. Stress during grain filling reduces both grain size and grain quality [69]. Sequential stresses spanning reproductive development, such as heat at anthesis followed by drought during grain filling, have more than additive effects, because reduced grain set constrains source–sink relations later [38]. Table S2 catalogues the principal climate-relevant compound stresses of wheat together with their stage-specific consequences and evidence strength. Interactions at reproductive stages are frequently synergistic, which argues for stage-specific engineering, although the available evidence does not establish that synergism is the rule at all reproductive stages [70,71,72].

These outcomes are governed by the interaction of four variables rather than by any one of them acting alone: genotype, the particular combination of stresses, stress intensity and developmental stage. The same mechanism can be beneficial in one window and detrimental in another; stomatal closure conserves water during vegetative growth, but at anthesis it restricts transpirational cooling and can aggravate heat-induced damage to floret fertility [55,56,57]. The effect of an edited or introgressed allele is therefore conditional on genetic background, phenology, root-system architecture and biomass allocation, so the yield consequence of a given construct cannot be inferred from its behaviour in a single background or at a single stage [9,38,41] (W/C/M). It is this interaction structure, rather than the identity of individual genes, that makes compound-stress resilience difficult to engineer.

## 4. Molecular and Physiological Trade-Offs Constrain Yield Resilience

### 4.1. Water Conservation Versus Transpirational Cooling

The classical drought response is ABA-regulated stomatal closure, a long-standing breeding target [73]. When heat accompanies drought, the same closure prevents transpirational cooling, raising leaf temperature by 3–6 degrees Celsius above ambient and increasing damage to the photosynthetic machinery and to reproductive structures [57,74]. Genotypes whose stomata are less sensitive to drought maintain cooler canopies but deplete soil water faster, leaving them more vulnerable if drought persists [55,75]. Engineering will need either tissue-specific control that permits some transpiration while protecting reproductive structures, or the capacity to adjust stomatal behaviour according to the combined stress state rather than water status alone [76]. Level B evidence indicates that natural variation in stomatal response kinetics correlates with yield stability across environments, although the underlying alleles are not fully characterised and the optimal target remains undefined [77].

### 4.2. Protection Versus Carbon Acquisition

Under excess excitation energy, oxidative damage to photosystem II is limited by non-photochemical quenching, thermal dissipation and antioxidant systems [78], but sustained photoprotection reduces photosynthetic efficiency once stress is relieved [79]. The molecular basis of recovery kinetics from the photoprotective state is poorly described, although genotypes with faster recovery achieve higher yield under intermittent stress [80]. Constitutive overexpression of antioxidant enzymes can deliver protection under acute stress at the cost of photosynthetic capacity under moderate or favourable conditions [81]. Inducible or recovery-tuned photoprotection is more attractive but requires understanding of the switches that govern transitions between photoprotective states [82].

### 4.3. Root Investment Versus Reproductive Allocation

Larger, deeper root systems increase water capture under drought and nutrient capture under low inputs [83], but root construction requires carbon that competes directly with spike and grain development [84]. Where root growth has been increased by breeding, a negative relationship between root biomass and harvest index is commonly observed in favourable environments [84]. The optimal architecture depends on the timing of water availability: deep rooting benefits terminal drought but confers little advantage when drought follows waterlogging, because hypoxia has already compromised deep-root function [85]. Engineering root architecture for compound stress may therefore require developmentally and environmentally conditional regulation, or alleles conferring plasticity [86]. QTL for tiller number and root angle are associated with drought resistance in wheat at Level C, but their behaviour under heat and waterlogging is poorly studied [87].

### 4.4. Ion Homeostasis Versus Nutrient Acquisition

Sodium exclusion by HKT transporters and vacuolar sequestration by NHX antiporters are the key salinity-tolerance mechanisms [36]. The cation selectivity filters responsible for exclusion can also impair potassium uptake, and increased vacuolar sequestration competes with potassium and calcium homeostasis [88]. When salinity and drought coincide, sodium-excluding genotypes may conserve water more effectively but accumulate higher leaf sodium because transpirational flux is reduced [37]. Combined heat and salinity adds complexity: sodium exacerbates heat-induced oxidative damage, and heat alters membrane transporter kinetics [36]. Level B evidence indicates that the yield benefit of enhanced HKT1;5-mediated exclusion appears only above threshold salinity, and is more pronounced when salinity is combined with low potassium availability in the field [89].

### 4.5. Stay-Green Versus Remobilization

Delayed senescence (stay-green) extends photosynthesis during grain filling and helps maintain assimilation under terminal stress [90], but can delay nitrogen and carbon remobilization to the developing grain, lowering protein content and harvest index [91,92]. Under combined heat and drought during grain filling the optimal strategy is severity-dependent: slow senescence under moderate stress, rapid senescence under severe stress to remobilize reserves before the canopy fails [93,94]. NAC transcription factors controlling wheat senescence timing are plausible targets, but direct manipulation risks mismatching remobilization to the stress actually experienced [95]; stage-specific or stress-responsive senescence control may resolve this [96].

### 4.6. Constitutive Defence Versus Context-Dependent Resilience

Constitutive activation of stress signalling, for example by overexpressing DREB, NAC or WRKY transcription factors, increases survival under severe stress in controlled environments [13] but imposes a well-documented growth penalty under favourable conditions [12,97]. The mechanism is twofold: the energetic cost of proteins and metabolites that are not required, and direct repression of growth by stress-signalling components that antagonise growth pathways. The classic demonstration is in *Arabidopsis*, where constitutive 35S-driven DREB1A conferred tolerance but caused severe growth retardation, whereas the stress-inducible rd29A promoter preserved tolerance while largely avoiding the cost [98]; rd29A-driven DREB1A in wheat likewise delayed water-stress symptoms without the constitutive penalty, although field yield was not assessed [99] (C). The same pattern holds when ABA signalling is constitutively activated or when osmolyte and antioxidant defences are elevated constitutively [100,101]. Stress-inducible promoters, tissue-specific expression and tunable regulatory elements can in principle escape these trade-offs, but they require an understanding of promoter structure and of homoeologue-specific control in wheat that is not yet available [102].

Figure 2 integrates these six trade-offs and shows that the balance point of each shifts with stress architecture, developmental stage and environmental context, so that no single point of intervention delivers yield-stable resilience [103,104]. Table 1 sets out the same trade-offs with their mechanistic bases, stress contexts and candidate mitigation strategies [105,106]. In every case, constitutive interventions carry costs incurred under conditions other than those in which they confer benefit; matching the intervention to the target climate is therefore essential, because what suits chronic drought may be counterproductive where waterlogging alternates with drought [107]. This context dependence is the core of our argument that tunable, context-responsive strategies are required [20].

## 5. Stress Memory as an Engineering Layer

### 5.1. Mechanistic Layers of Memory

Stress memory is a persistent biological state that modifies subsequent stress responses as a result of prior exposure [16]. This distinguishes memory both from an immediate physiological response that decays once stress is relieved and from persistent injury, which reflects incomplete recovery rather than regulated preparedness [47]. Several mechanisms can retain stress information across a recovery interval [15] (M). Chromatin modifications alter transcription upon re-exposure, including retention of trimethylated H3K4 (H3K4me3) at primed stress-responsive genes and altered H3K27me3 dynamics at developmental genes; most such evidence comes from *Arabidopsis*, and causal demonstration in wheat is rare [112,113,114,115,116] (C/M). DNA methylation is remodelled under drought and heat, but these changes are not stable across development or generations because of active and passive demethylation during reproduction [117] (C).

Primed states can also be sustained by persistent transcription-factor activity, for example by autoregulatory loops involving heat-shock factors or dehydration-responsive element-binding proteins [118]. Small RNAs, including stress-induced microRNAs and small interfering RNAs, have been proposed to propagate systemic signals and to help maintain memory states, although functional validation in wheat is limited [119]. Biochemically direct somatic memory reflects the persistence of metabolites or proteins, such as maintained osmolyte pools, antioxidant capacity or heat-shock protein levels; its decay time matches the turnover half-life of the protein or metabolite concerned [120,121]. Integrative memory layers, in which hormonal or redox states are altered, notably through sustained ABA elevation and retained ROS-scavenging capacity, condition whole-plant responsiveness [61,122].

### 5.2. Evidence Required to Establish Causality

A claim of stress memory must satisfy a demanding evidential ladder [123]. Transcriptional change following re-exposure establishes association, not memory. The criteria are: (1) persistence of a molecular or physiological state beyond the immediate removal of stress; (2) a response to re-exposure that differs from that of stress-naive controls; (3) causal perturbation of the proposed memory mechanism with a correspondingly altered response to re-exposure; and (4) reproducibility across environments and genotypes [25]. Where an intergenerational or transgenerational origin is claimed, two further criteria apply: (5) evidence in unexposed offspring, with maternal, paternal and environmental controls that exclude seed provisioning effects and residual environmental variation; and (6) persistence across multiple unexposed generations, which is required for true transgenerational inheritance [26,124,125].

Most wheat studies reach only the first two steps [126,127]. Although chromatin modifications change under stress, few studies have manipulated chromatin modifiers to establish causality [128], and many apparent transgenerational effects are more plausibly explained by maternal seed provisioning or environmental carryover [27,129]. Cross-stress memory adds complexity: exposure to one stressor may enhance tolerance to another through shared ABA and ROS pathways, but the converse is equally plausible [130,131].

Figure 3 sets out these mechanistic layers alongside the evidential staircase, distinguishing the stages at which stress memory can be established rather than merely correlated. It is intended as a framework for judging memory claims and for determining the experimental evidence required before memory-based engineering is deployed [132,133].

### 5.3. Opportunities and Liabilities of Engineered Memory

Unlike constitutive stress activation, engineered memory offers a potentially favourable cost profile [134,135,136]: a primed plant grows normally under favourable conditions but mounts a faster or stronger defence upon exposure [137,138]. Candidate approaches include promoter editing to render chromatin stress-responsive, CRISPR activation and interference at memory-associated genes, epigenome editing to deposit primed marks, and synthetic circuits built on toggle-switch or feedback architectures [139,140] (M/W). Substantial liabilities remain [141]: memory can vary unpredictably between contexts, be reset at developmental transitions and carry cryptic metabolic costs [142,143]. Maladaptive memory, in which a past stressor primes a response to a stress that never returns or that differs from the original, may degrade performance under multi-stress conditions, and distinguishing beneficial priming from continuing injury is experimentally difficult but essential [144].

The persistence and reproducibility of memory are not fixed properties of a mechanism but depend on stress intensity and duration, the developmental stage at which priming occurs, the genotype and the environment in which the second exposure is delivered [124,125,127,129]. Intergenerational and transgenerational effects in wheat are frequently environment-dependent and may weaken or disappear when offspring are grown under different conditions [25,26,27]. Two consequences follow. First, a memory mechanism demonstrated in one environment cannot be assumed to operate in another; stability across environments and genotypes must be tested rather than inferred. Second, the distinction between memory mechanisms that have been demonstrated and engineering opportunities that have merely been proposed must be kept explicit, because the two currently rest on evidence of very different strength [142,143,144].

Table S3 assesses each memory layer for its evidential foundation and engineering readiness, and the gap it reveals is stark. Although several memory mechanisms have been described in wheat and other species, causal validation by genome editing followed by field testing is absent throughout. To our knowledge there is no field-tested, genetically validated wheat stress-memory mechanism, that is, none at evidence Level A or B, on which a current memory-engineering proposal could be based; all remain at Level D or E [31,145,146].

## 6. Molecular Targets for Yield-Stable Compound-Stress Resilience

The targets reviewed below are organised by functional module rather than by gene family, from signal perception and integration (Section 6.1) through hydraulic, ion, osmotic, proteostatic, reproductive and chromatin-related processes (Section 6.2, Section 6.3, Section 6.4, Section 6.5 and Section 6.6), because resilience to compound stress depends on their coordinated regulation rather than on any single node [147].

### 6.1. Stress Perception and Signal Integration

Signal-integration nodes that determine the response to multiple stresses are higher-value targets than terminal effectors responding to a single stress [147]. The core drought-signalling module comprises the ABA receptors (PYR/PYL/RCAR), their interacting PP2C phosphatases and SnRK2 kinases, components of which are also implicated in heat and salinity crosstalk [148]; each exists as several homoeologues with similar but not identical functions [149]. The engineering challenge is calibration: excessive ABA sensitivity over-reduces stomatal conductance, restricting growth and lowering yield under combined heat and drought, whereas insufficient signalling impairs water conservation [150]. Promoter manipulation of specific PP2C or PYL homoeologues, altering guard-cell ABA sensitivity without disturbing reproductive ABA responses, is theoretically attractive but unexplored [151].

Calcium signatures are decoded by calcium-dependent protein kinases (CDPKs) and by calcineurin B-like (CBL) sensors acting with the CIPK family [152]. Wheat CDPKs are differentially activated by drought, heat and their combination, with some isoforms activated only under combined stress [63]. MAPK cascades act as universal signal-transduction modules of plant stress responses, and stress-responsive MAPK and MAPKK family members have been identified genome-wide in wheat [153]. These nodes are poorly quantified but are candidates for engineering altered stress prioritisation, for instance by biasing the stomatal MAPK pathway to respond preferentially to heat rather than drought when both are present [154].

### 6.2. Stomatal, Hydraulic and Root-System Regulation

Guard-cell-specific engineering could uncouple water conservation from leaf cooling [76]. In wheat this is complicated by multiple homoeologues of both stomatal development genes (SPCH, MUTE, FAMA) and guard-cell ion channels (SLAC1, GORK, KAT1), which must be manipulated in calibrated combinations. Reducing stomatal density in bread wheat increases water-use efficiency (WUE) [76], and comparable results in barley indicate improved drought tolerance without a yield penalty, suggesting that the WUE–carbon trade-off may be more tractable than assumed [111] (C). Root and leaf hydraulics are regulated by PIP and TIP aquaporins; certain PIPs are downregulated by drought yet maintained or upregulated under combined drought and heat in some cultivars, offering scope to engineer sustained hydraulic function [155]. QTL for deep rooting, root angle and lateral root density have been mapped, but their interaction with above-ground stress responses and the contributions of individual homoeologues remain poorly characterised [87].

### 6.3. Ion, Osmotic and Redox Homeostasis

The major wheat salinity locus HKT1;5 on chromosome 4D has been confirmed in the field [89], and gene families are available for sodium transporters (HKT1;4, HKT1;5, SOS1) and vacuolar Na+/H+ exchangers (NHX1-NHX5). Under combined stresses, ion-homeostasis requirements change substantially [88]: salinity with drought requires both exclusion and water conservation, which may favour a different HKT expression level than salinity alone [37], whereas salinity with heat may require greater vacuolar sequestration to protect cytoplasmic enzymes while maintaining potassium uptake for reproduction [36]. Osmotic adjustment (the accumulation of compatible solutes that maintains turgor and protects macromolecules as water potential falls) is the third component named in the title of this section and the least developed for combined stress. Combined heat and drought alters cell osmoregulation alongside photosynthesis and nitrogen metabolism in wheat [42], but the benefit is carbon-dependent: the solutes that provide osmotic protection draw on the same assimilate pool that grain filling requires, so the net outcome depends on the stage at which stress occurs. Redox homeostasis is attractive precisely because ROS act both as damaging agents and as essential signals [64]. Wheat peroxidases, catalases, superoxide dismutases and glutathione–ascorbate cycle enzymes show homoeologue-specific regulation under combined stress [108]. Engineering must limit oxidative damage without eliminating the ROS signals required for acclimation, an outcome constitutive overexpression cannot achieve [81]; stress-inducible or compartment-restricted approaches offer greater precision [108].

### 6.4. Proteostasis, Photosynthesis and Energy Balance

Heat-shock proteins (HSP70, HSP90 and small HSPs) and their regulatory heat-shock factors (HSFs) maintain proteostasis during heat stress and show functional specialisation among wheat homoeologues [109]. Drought imposes both proteotoxic and energetic burdens, and HSP induction requires ATP that drought-stressed plants may be unable to supply [110]; engineering HSP expression must therefore consider induction strength, energy availability and recovery capacity [118]. Rubisco activase, a heat-labile enzyme essential for carbon fixation, is a thermotolerance target via both transgenic and natural-variation routes [156] (C); wheat isoforms differ in heat sensitivity, and greater thermostability must not compromise efficiency at the intermediate temperatures at which most grain filling occurs [157]. Trehalose 6-phosphate (T6P) signalling links carbon status to growth and development; editing trehalose phosphate synthase (TPS) or trehalose phosphatase (TPP) genes can alter grain size and stress response, but constitutive manipulation disrupts normal development [158].

### 6.5. Reproductive Protection and Source-Sink Regulation

Maintaining reproductive development under compound stress is a priority, because spike fertility and grain filling translate directly into yield [9]. Stress disrupts pollen development, which requires a specific sugar, lipid and hormonal environment [159]. NAC and MADS-box transcription factors controlling floret fertility and grain number are candidates, but homoeologue redundancy and pleiotropic developmental roles complicate manipulation [95]. Source-sink coordination becomes critical when stress reduces the photosynthetic source during grain filling [160]: SWEET and SUT transporters mediate assimilate delivery, and several SWEET genes are downregulated under drought and heat in wheat, contributing to grain shrivelling [161]. Engineering resilience here requires sustained transporter activity under stress without perturbing source-sink balance under favourable conditions [162].

### 6.6. Chromatin and Memory-Associated Targets

Chromatin modifiers constitute a high-risk, high-reward class of targets [163] (R). Histone deacetylases and acetyltransferases regulate stress-responsive expression, and certain HDACs have been implicated in drought and salt tolerance in rice [113] (C). DNA methylation is established and remodelled by methyltransferases and demethylases, but methylation-mediated memory has not been causally demonstrated in wheat [117] (C). Polycomb deposition of H3K27me3 controls developmental transitions and stress responses, so modulating its dynamics would be expected to shift the growth–defence balance [112]. Because chromatin modifiers influence hundreds to thousands of loci, such pleiotropy demands precise targeting—for example, dCas9 fusions directed to specific stress-responsive loci rather than global perturbation [139].

Figure 4 relates the A, B and D subgenomes to target tissues and molecular modules, and illustrates a prioritisation framework that integrates causal evidence, agronomic benefit and engineering feasibility [164,165]. Table S4 converts this landscape into a target–tool matrix, listing for each biological module the preferred editing strategy, relevant homoeologue-dosage considerations and current readiness [166,167]. Most targets sit at evidence Level C or lower, which measures the distance that still separates mechanistic understanding from practice-ready engineering.

## 7. Precision Engineering of the Hexaploid Wheat Genome

### 7.1. Homoeologue Dosage as an Engineering Variable

Homoeologue dosage is an engineering variable with no counterpart in diploid crops [168]. Most wheat genes occur as triads on the A, B and D subgenomes, and these copies differ in expression level, tissue specificity and stress responsiveness [169]. Editing one, two or three copies therefore generates a phenotypic gradient rather than an all-or-nothing outcome [170], which is most valuable where complete knockout would be lethal or severely pleiotropic. Triple-homoeologue knockout is appropriate where complete loss of function delivers a clear benefit, as achieved for the wheat MLO genes in powdery mildew resistance [171,172]; where a gene is stress-regulated and a strong response is desirable only in particular contexts, partial dosage may outperform complete manipulation [173]. Design is complicated by homoeologue expression dominance, in which subgenome copies contribute unequally to total expression [174,175,176], so systematic characterisation of expression bias under compound stress is a prerequisite for rational dosage design [174,175] (W/C). Comparable partitioning of homoeologue expression among subgenomes has been documented in other polyploid crops [177].

The 0–3 homoeologue editing framework is a heuristic device that enumerates the design space, and it should not be read as implying a predictable linear relationship between the number of edited copies and the phenotype. Four factors modulate that relationship. First, homoeologue expression bias: the three copies are not equally expressed at baseline, so the effect of knocking out any one copy depends on that copy’s original contribution [168,175]. Second, compensation: the remaining copies may be upregulated and partially offset the loss. Third, tissue and stage specificity: the same copy contributes differently in different organs and at different developmental stages, so copy number cannot be interpreted independently of expression context [176]. Fourth, genotype × environment interaction: a given dosage may be beneficial in one environment and neutral or deleterious in another [178]. The framework is therefore best understood as a way of enumerating the space of possible designs and requiring each to be tested, rather than as a means of predicting the outcome of an edit.

### 7.2. Choosing Between Knockout, Allelic and Regulatory Editing

The approach should match the biological aim [179,180]. Triple-homoeologue knockout with Cas9 or Cas12a is appropriate only where loss of function delivers benefit without pleiotropic cost, rarely the case for stress-regulatory genes also required for development [181,182]. Allelic replacement by base or prime editing introduces precise nucleotide changes that recreate beneficial natural alleles [183]: stress-tolerance alleles at NAC, DREB and HKT loci differing from elite alleles by single amino acid substitutions, which would otherwise require introgression from wild relatives, can in principle be recreated directly in elite germplasm without linkage drag [184,185,186,187,188,189]. Cytosine and adenine base editors are active in wheat but constrained by PAM availability, a narrow editing window and bystander editing, which must be managed through guide RNA design and event screening [184].

Promoter and cis-regulatory editing is the best option for tuning stress responses while preserving essential gene function [190,191]. Wheat promoters contain stress-responsive elements, tissue-specific enhancers and developmental switches that can be modified to alter the timing, magnitude or tissue specificity of expression [192]; editing ABA-responsive elements (ABREs) can alter ABA sensitivity, and editing dehydration-responsive elements (DREs) in DREB target promoters can alter their activation [193]. Promoter function nonetheless depends on combinatorial transcription-factor binding that is poorly predicted from the sequence alone, and wheat promoter annotation lags well behind rice and *Arabidopsis* [194]. Fusing dCas9 to transcriptional effectors enables reversible control through CRISPR activation (CRISPRa) and CRISPR interference (CRISPRi) without altering the sequence [195]; wheat CRISPRa/i systems are functional but variable in efficiency and require sustained dCas9-effector expression, so they are transgenic and would be regulated as such in some jurisdictions [196].

### 7.3. Emerging Programmable and Synthetic Approaches

Prime editing enables insertions, deletions and all possible base substitutions without double-strand breaks or donor templates [197]. Early reports in wheat placed its efficiency at 1–10%, but subsequent improvements in prime-editing guide RNA design, editor architecture and delivery have raised achievable efficiencies, including in multiplex formats in hexaploid wheat [198,199,200,201] (W/C). Specific efficiencies should therefore not be treated as fixed criteria. The operative point is that prime editing in wheat remains appreciably less efficient than nuclease or base editing, and that its efficiency depends strongly on the target site, genotype and transformation system, which is presently the principal technical constraint on its use where several homoeologue copies must be edited simultaneously. Chromatin states at stress-responsive loci can be set directly by fusing dCas9 to DNA methyltransferases, demethylases or histone-modifying enzymes, collectively termed epigenome editing [202,203], although wheat epigenome editing remains proof-of-concept (M). Resilience strategies might ultimately be implemented as synthetic circuits in which a stress-inducible promoter is coupled to logic-gate processing and an effector output [204], or by pyramiding multiple tolerance loci through marker-assisted selection [205]; the engineering of synthetic regulatory circuits in plants more generally remains an area of active development [206]. Such multi-component systems face regulatory, stability and breeding hurdles that currently confine them to conceptual investigation.

### 7.4. Delivery, Regeneration and Molecular Quality Control

Editing and regeneration remain among the largest bottlenecks in wheat [207]. Immature-embryo transformation by *Agrobacterium tumefaciens* or biolistics is strongly genotype-dependent, with low regeneration efficiency in most elite cultivars, although GRF-GIF chimeric constructs have improved regeneration [208,209]. Pre-assembled Cas9-guide RNA ribonucleoprotein (RNP) complexes support DNA-free editing and work in wheat protoplasts and immature embryos, but routine RNP editing across diverse germplasm has not been achieved [210]. Tissue-culture-free approaches, including viral delivery and meristem transformation, remain developmental [211]. Tissue culture also introduces somaclonal variation that confounds stress phenotypes and necessitates multiple independent events and backcrossing to clean backgrounds [207]. Molecular quality control should verify on-target edits in every targeted homoeologue, phase A, B and D copy edits, detect on-target rearrangements, screen predicted off-target sites and confirm the absence of transgenes where required [212]; in current practice one or more of these steps is routinely omitted, leaving genotype–phenotype relationships unclear [213]. Figure 5 couples the choice of editing tool to homoeologue-dosage decisions and to this quality-control pathway, emphasising that the nature of the intended biological change should determine the tool selected and highlighting the specific constraints that apply in hexaploid wheat [214].

### 7.5. Germplasm Resources and Breeding Integration for Compound-Stress Resilience

Domestication and improvement bottlenecks mean that the cultivated wheat gene pool retains only part of the allelic variation present in its wild relatives [164,165,188]. Wild and exotic gene pools—*Aegilops tauschii*, wild emmer (*Triticum dicoccoides*) and *Thinopyrum species*—preserve stress-adaptive alleles that were lost or never incorporated [188,189]. Introgression lines from *A. tauschii* improve drought and heat tolerance and yield-related traits in bread wheat [186,187], and a global panel analysis reveals a diverse introgression landscape with inter-homoeologue recombination [189] (W/C).

Three constraints govern the practical use of introgression lines. First, linkage drag: the donor segment carrying the target allele also carries linked alleles that are frequently unfavourable, and dissociating the two requires extensive backcrossing and recombination screening [187,189]. Second, suppressed recombination: recombination between alien and cultivated chromatin is often depressed, limiting fine dissection of the introgressed segment [189]. Third, background dependence: the phenotypic effect of an introgressed segment varies with genetic background, so an allele advantageous in one background may be neutral or deleterious in another. These constraints do not diminish the value of introgression, but they define what it can deliver (W/C).

Introgression and precision editing consequently occupy different positions in a single pipeline rather than competing as alternatives. Introgression introduces allelic variation that has already survived natural selection, whereas editing deploys a defined allele in an elite background with minimal linkage burden [184,190,191] (W). Where a favourable allele differs from the elite allele by one or a few nucleotides, base or prime editing can recreate it directly [184,190]; where the causal variant is unknown, or the trait depends on many loci, introgression remains the only route [186,187]. Editing is therefore treated here as the principal engineering route, with introgression as an indispensable upstream source of variation.

At the population level, recurrent selection accumulates alleles of small individual effect while maintaining genetic variation [215]. Stress tolerance is typically polygenic: individual loci explain little phenotypic variance and are easily masked by environmental noise, so single-cycle selection is inefficient, whereas repeated cycles of recombination and reselection raise the frequency of favourable alleles progressively [215,216] (W/C). Marker-assisted pyramiding applies the same logic to defined loci and has been used in wheat to combine rust resistance, grain protein content and pre-harvest sprouting tolerance within single genotypes [205], now complemented by genomic prediction, which shortens cycle time while retaining the multi-locus character of the trait [215,216]. The costs are a long cycle and the need for an adequately large effective population size to avoid genetic drift.

A more speculative proposal is to select across successive segregant generations on the basis of primed performance: a standardised priming treatment would be applied to a segregating population each generation, and responsive individuals retained, assuming heritable priming capacity can be accumulated like any other trait [124,125]. This must be presented as a research agenda rather than a validated method. Intergenerational and transgenerational drought effects have been mapped in wheat [124,125,127,129], and drought priming alters grain starch and protein quality under grain-filling stress [135,136] (W); but the distinction between heritable memory and non-heritable maternal or seed-provisioning effects is rarely resolved, and the causal status of transgenerational memory in wheat remains unsettled [25,26,27]. Until reciprocal-cross designs exclude maternal and seed-provisioning effects and establish persistence across unexposed generations, priming performance cannot serve as a selection criterion.

The same logic applies to the upstream identification of targets. Wild relatives and the wider cultivated gene pool, including landrace diversity within the primary gene pool, are the source of alleles that domestication and modern breeding have narrowed or excluded [164,189]. Pre-breeding programmes and introgression pipelines convert that variation into material that can be evaluated and, where a favourable allele differs from the elite allele by one or a few nucleotides, recreated directly by editing [186,187,188]. Physiological breeding, in which selection is based on traits that integrate stress response rather than on yield alone, and genomic prediction, which shortens the selection cycle while retaining the multi-locus character of stress tolerance, both operate before or alongside editing by identifying and validating which alleles are worth manipulating [10,215,216,217]. Editing is therefore best understood as a complement to, rather than a replacement for, natural variation and conventional breeding; the two act at different points in the same pipeline.

The integration is therefore sequential rather than additive. Wild relatives supply allelic variation; precision editing rebuilds or fine-tunes a target allele in an elite background free of linkage drag; recurrent selection or marker-assisted pyramiding aggregates small-effect loci at the population level; and multi-environment trialling (Section 8.4) tests whether the resulting lines remain stable where they are to be grown. This division of labour, rather than any single technology, is what compound-stress resilience in wheat is most likely to depend on [10,217].

### 7.6. Combining Stress-Tolerance Genes: Cross-Stress Trade-Offs and Design Constraints

Section 4 examined the trade-offs intrinsic to individual protective traits. This section addresses a different question: what happens when several individually beneficial interventions are combined in one genotype? The answer cannot be obtained by summing the single-gene effects, because the outcome is governed by the architecture of stress signalling rather than by the properties of any individual gene.

The first class of interaction is non-additivity, or epistasis. Two edits that each improve performance in isolation may combine sub-additively, additively or even antagonistically, and the outcome is least predictable when both targets lie within the same signalling pathway [53,54,62]. Enhancing ABA-mediated stomatal closure while simultaneously enhancing antioxidant capacity may be self-defeating, because the first response reduces transpirational cooling whereas the second protects against the oxidative consequences of the resulting heat load [55,57]. Crosstalk among ABA, jasmonate, salicylic acid, ethylene and cytokinins provides many such junction points [29,62]. Quantitative study of these interactions in wheat is scarce, and most available inferences are drawn indirectly from single-gene experiments, so they cannot be used predictively (C/M).

The second class arises from homoeologue dosage. The number of homoeologue copies that must be edited rises with the number of target loci, increasing both the probability of unintended edits and the risk of chromosomal rearrangement [170,173]. More fundamentally, the phenotype associated with a given dosage is not fixed: homoeologue expression bias, compensation among remaining copies, tissue and stage specificity, and genotype × environment interaction all modulate it [174,175,176]. As argued in Section 7.1, copy number cannot be interpreted independently of the expression context, and the more loci are stacked the less predictable the aggregate dosage effect becomes [174,175] (W/C).

The third class is the stacking of costs. Individual constitutive defensive traits each impose a penalty under favourable conditions, and the combined penalty of several can exceed the sum of the individual penalties, because the resource pools on which they draw—carbon, nitrogen and energy—are shared and their allocation is non-linear [12,67,105]. When several defence pathways compete for the same limited pool, the marginal cost of each additional pathway rises; and where two traits make opposing demands on the same resource, such as when increased root investment competes with spike development for assimilate, the combination may perform worse than either trait alone [83,84,106]. Growth–defence allocation is coordinated through SnRK1 and TOR signalling, so interventions that shift this balance do not act independently [67]; transcription factors coupling stress responses to developmental programmes illustrate the same entanglement [97].

Specific stress pairs show that direction, not merely magnitude, can change. Under combined salinity and drought, ion exclusion and water conservation impose opposing requirements on HKT expression, and the optimal level differs from that for either stress alone [37,88]. Waterlogging followed by drought is a second case: the root traits that favour survival of hypoxia are not those that maintain water capture once the soil dries [44,85]. Combined heat and salinity adds a third: sodium exacerbates heat-induced oxidative damage while high temperature alters membrane transporter kinetics, so the protective requirement shifts with the combination [36]. Combinations involving salinity and nutrient limitation behave similarly, because the energy cost of ion homeostasis competes with nutrient acquisition [88] (C).

Three design principles follow. First, stress-inducible or tissue-specific regulatory elements should be preferred to constitutive ones, confining the cost of the intervention to the time and organ in which stress occurs [98,99,103]. Second, signal-integration nodes should be preferred to terminal effectors where feasible, because a single change at a node can mobilise several downstream pathways and cover a broader stress spectrum with fewer edits—at the price of greater pleiotropic risk, which must be assessed rather than assumed [147,148,152,218]. Third, the number of stacked targets should be escalated stepwise with verification at each level, rather than combining all edits before phenotyping [170,190].

The quantitative evidence for these interactions in wheat is very limited. Most of what is known derives from other cereals and from model species, or from indirect comparison across single-stress experiments, and this constrains the predictive power of the present discussion (C/M) [53,54,62]. That gap is itself the argument for the position taken in Section 8: because the outcome of stacking cannot be predicted from single-gene behaviour, it must be measured under realistic field conditions rather than inferred, and it is accordingly listed among the research priorities in Section 10 [178,219].

## 8. From Edited Plants to Field-Level Yield Stability

### 8.1. Designing Biologically Realistic Stress Tests

A recurring failure point is the gap between controlled-environment phenotyping and field performance [220]. Single-stress protocols that apply continuous stress throughout the vegetative phase and score survival or biomass systematically overestimate field translatability [219]. Realistic testing must instead reflect the temporal architectures described in Section 3, addressing the stress regime (concurrent stresses at agriculturally relevant stages), sequence (discrete stresses separated by ecologically realistic intervals), recurrence (repeated events of defined frequency and duration) and recovery (assessed as a component of resilience rather than survival). Intensity should be calibrated to the target population of environments, not to levels that eliminate all genotypes [178,218].

Stress is not a binary treatment. The outcome of a combined stress depends jointly on the intensity and duration of each component, their frequency, the stage and rate at which they develop, the recovery interval that follows, soil water status, vapour pressure deficit, night-time temperature, radiation and their interaction with the developmental stage [35,38,43,178]. A protocol that fixes these variables at a single extreme value samples one point from a space of biologically plausible trajectories, and seldom the point that crops encounter. Trials should therefore aim to reproduce realistic stress trajectories, including the rate of imposition and the length of the recovery interval, rather than maximal stress, so that the mechanism tested is the one that operates in the field, where yield stability is ultimately decided [49,50,219,221].

Glasshouses and growth chambers permit controlled stress imposition but cannot reproduce the diurnal temperature and humidity dynamics, unrestricted soil volume and light quality gradients that shape transpiration, root function and canopy temperature [221]. Field-based stress imposition using rainout shelters, heat tents and managed-stress environments provides more realistic conditions but introduces spatial and temporal variability that demands careful experimental design [51]. A staged approach is therefore necessary, moving from controlled single-stress screening to managed compound-stress testing, and then to multi-location field testing [222].

### 8.2. Measuring Resilience Rather than Survival

Resilience must ultimately be quantified as the grain a farmer harvests and the stability of that harvest across environments [11]. Several quantities that are frequently conflated must be distinguished: mean yield, the expectation of yield across the environments of interest; stress yield, yield under a defined stress scenario; yield penalty, the difference between the edited line and its isogenic control under favourable conditions, usually expressed as a percentage; environmental sensitivity, the slope of the response of yield to an environmental index, as in the Finlay–Wilkinson regression coefficient; and yield variance, the dispersion of yield across environments, or more formally a stability variance such as Shukla’s stability variance or Wricke’s ecovalence [222,223]. Beyond final yield, informative intermediate phenotypes include canopy temperature dynamics (stomatal regulation and cooling capacity), gas exchange (photosynthetic performance during stress and recovery), root architecture and hydraulic traits (water acquisition), ionome analysis (nutrient imbalance), pollen viability and spike fertility (reproductive protection), thousand-grain weight and grain dimensions (harvest success), harvest index (allocation efficiency), and grain protein and starch composition (end-use quality) [224,225,226,227] (W/C).

Yield stability is defined in this review as low temporal and spatial variation in yield across the target population of environments, combined with a mean yield that is not significantly below that of the appropriate check. Two points are essential. First, stability is a property relative to a defined population of environments; without that definition, stability statistics have no interpretable meaning [178,222]. Second, stability must be evaluated jointly with mean yield, because a genotype of uniformly intermediate performance can be statistically very stable while being agronomically inferior to a genotype that performs well in most environments and declines only in extreme years [107].

Recovery must be measured explicitly after stress relief, because genotypes that survive without resuming growth and grain production have no agronomic value [46,228], and performance under favourable conditions must be tested rather than assumed [12,107,229]. Yield-stability statistics, including genotype-by-environment interaction analysis, Finlay–Wilkinson regression and AMMI or GGE biplot approaches, quantify stability but require multi-environment datasets substantially larger than those typical of proof-of-concept studies [223,230,231,232,233,234]; the design and interpretation of such trials are set out in Section 8.4.

### 8.3. Multi-Environment Validation and Decision Gates

We recommend an evidence-gated validation pathway with explicit go/no-go criteria (Table 2). Molecular validation requires correct edits in the targeted homoeologues, no unintended rearrangements, defined expression changes and no off-target edits at predicted sites [235,236]. Controlled-environment tests must then be conducted at agriculturally relevant stages and intensities, under both single and compound stress, demonstrating protection or improved recovery without significant penalty relative to isogenic controls under favourable conditions [237,238]. Managed-environment or field testing follows, using realistic stress architectures that include recovery periods and recurrent events, independently derived events and measurement of yield components and grain quality. Multi-location, multi-season trials in target production environments and locally adapted backgrounds then confirm yield stability and the absence of unacceptable performance in any environment. Deployment gates address biosafety, regulatory approval, intellectual property and integration into breeding programmes, with stringency proportionate to the deployment target [239,240].

Table 2 specifies, for each of ten development gates, the minimum experimental design, essential controls, measurements, pass criteria, failure warnings and reporting requirements [245]. The explicit go/no-go criteria are intended to ensure that edited wheat lines advance from target selection to deployment only on the strength of evidence, and specifically to prevent weak controlled-environment phenotypes from being promoted as deployment-ready climate resilience [246].

The numerical criteria in Table 2—the 10% favourable-condition penalty at Gate 3, the 15% failure warning, and the minimum of three locations and two seasons at Gate 6—are illustrative thresholds proposed by the authors, not generally accepted standards, and they are not calibrated against empirical data. They show how decision criteria can be made explicit; individual programmes should set their own thresholds according to the target population of environments, the trait concerned and their risk tolerance [178].

Four senses of significance must be separated when such thresholds are applied. Statistical significance concerns whether an effect exceeds sampling error and depends on design and replication; biological significance is whether the effect alters a physiological process; breeding value is whether it can be selected reliably in a segregating population with adequate heritability, and deployment relevance concerns whether it is large enough to change adoption decisions under producers’ cost and risk constraints. An effect may be statistically significant yet of no breeding value, or biologically significant yet not statistically significant because replication was insufficient [219,235]. The thresholds in Table 2 approximate the latter three senses and are not critical values for statistical testing.

The preceding discussion treated genotype × environment interaction as the principal complication, but several traits considered here are also strongly modified by management and must therefore be understood as genotype × environment × management (G × E × M) interactions. Stomatal conductance responds to planting density, irrigation regime and nitrogen supply, which alter canopy microclimate and hence the direction of the effect of stomatal traits [226,227]; root architecture responds to tillage, sowing date and soil compaction, and the measurability of root phenotypes itself limits estimation of G × E for root traits [86,87]; and stay-green and source–sink regulation respond to nitrogen supply and sowing date, with stay-green potentially increasing yield under nitrogen limitation while delaying maturity and reducing harvest index where nitrogen is adequate [91,93,106,161]. For edited lines involving these traits, management practices must therefore be recorded and entered as covariates in the analysis, as developed in Section 8.4.

Linking climate risk [247,248] to yield-stable edited wheat requires a stage-gate process with explicit decision criteria, spanning mechanistic understanding, calibrated editing, realistic stress testing and responsible deployment; without it, the translation from mechanism to field-level yield stability will not occur.

### 8.4. Multi-Environment Field Trials for Wheat Yield and Stability Under Stress Environments

Compound-stress effects are strongly environment-specific: the same genotype may encounter different temporal stress structures at different sites and consequently express different yield responses [35,38]. A single-location trial cannot estimate genotype × environment interaction (G × E) and therefore cannot answer the question that defines this review, namely whether a line is stable in production. Field validation is consequently not a confirmatory step that follows molecular validation; it is the test on which the engineering strategy stands or falls [219,220]. The gap between controlled-environment performance and field performance is systematic rather than incidental: controlled environments lack the diurnal temperature and humidity dynamics, unrestricted soil volume and light gradients that govern transpiration, root growth and canopy temperature, and they typically score survival or biomass rather than grain yield and its stability [219,221].

Four design requirements follow. Site selection must represent the target population of environments (TPE) rather than being random or convenient [222,223]: the TPE is defined by the distribution of stress scenarios actually encountered in the region of intended deployment, so where the dominant risk is concurrent heat and drought during grain filling, sites should be selected according to the probability of that scenario rather than for logistical ease. Otherwise the estimated stability describes an environment population that does not exist. The realised stress structure must also be documented at every site—concurrent, sequential or recurrent—together with the intensity and duration of each component and the conditions prevailing during recovery intervals [35,38,43]. Without such records, trials cannot be assigned to the temporal architectures of Section 3.1 or compared across sites [49].

Both isogenic null-segregant controls and locally adapted check cultivars should be included. The former isolates the effect of the edit, whereas the latter establishes whether the resulting line is worth growing in that environment [11,178]; neither substitutes for the other. Management practices must finally be recorded and entered into the analysis as covariates, since otherwise the G × E estimate is confounded with management effects and cross-trial comparison becomes unreliable [216,222].

Any conclusion about stability depends on the statistic used to express it, and the principal families differ in what they measure and how they can be misread. Variance-based measures, of which Shukla’s stability variance and Wricke’s ecovalence are the most common, quantify how strongly a genotype’s yield fluctuates across environments [223,231]. Their comparative reading is not transportable: ecovalence in particular depends on the set of genotypes included, so a genotype’s value changes when trial composition changes and scores cannot be compared across experiments. Regression-based measures, principally the Finlay–Wilkinson joint regression, describe how strongly a genotype responds to environmental improvement through the regression coefficient and the deviation mean square [222,223]. A coefficient must always be read together with mean yield, since ranking on the coefficient alone systematically favours genotypes that are statistically stable but agronomically poor.

Multiplicative models, including AMMI and GGE biplots, describe the structure of the interaction itself and indicate which genotype performs best in which environment [222,230,231,232,233]. Their interpretation carries its own requirements: AMMI demands a defensible choice of the number of interaction principal components to retain, and the ‘which-wins-where’ reading of a GGE biplot requires a sufficient number of environments, since with too few the apparent pattern of the interaction is defined largely by noise [222,223]. Recent multi-environment analyses of wheat grain yield illustrate these approaches across stress and non-stress environments [230,231,232,234], although the number of environments in many published trials remains modest. The reliability of any of these statistics depends on the number of environments and years sampled: the minimum of three locations and two seasons proposed at Gate 6 in Section 8.3 is sufficient to show that an effect is not confined to a single environment, but is generally too small to estimate stability variance or the structure of the interaction with useful precision. Where the objective is to estimate yield stability rather than merely to detect an effect, the number of environments must be substantially greater, and environments should be sampled from the TPE rather than chosen for convenience [222,223]. Stability statistics reported from small trials should be described as exploratory.

A further distinction is easily lost: statistical stability is not agronomic yield stability. A genotype of uniformly intermediate performance may show small variance and a regression coefficient near unity, and is therefore highly ‘stable’ in the statistical sense, while being inferior to one that yields highly in most environments and declines only in extreme years; the latter has a higher mean yield and a larger variance [107,178]. Ranking on stability statistics alone therefore selects systematically against the higher-yielding genotype, so stability must be evaluated jointly with mean yield. Two practices are available: reporting mean yield and stability statistics side by side, making explicit that the weight assigned to each depends on the breeding objective [178], or using a composite criterion such as the ideal-genotype measure of a GGE biplot, which identifies the genotype simultaneously closest to the highest mean performance and to the most stable position [230,231]. This review recommends the first, because the weighting of risk should be set by the risk characteristics of the target environment population and the risk tolerance of those who will adopt the cultivar, rather than determined implicitly by the statistic chosen.

The design described here is the direct basis for Gates 5 to 8 in Section 8.3: Gate 5 supplies the estimate of the stress effect, Gate 6 the estimate of stability, Gate 7 the estimate of the yield and quality penalty, and Gate 8 the quality constraint. Together they answer the question emphasised throughout this review—whether maintenance of yield under stress is achieved at the cost of yield or quality under favourable conditions. If any one element is missing, the claim of yield-stable resilience is not supported [107,220].

These requirements must be reconciled with real constraints. Multi-environment trials are expensive, suitable sites are not always available, and multi-year trials suffer attrition through drought, disease and changes in land use; public-sector programmes in particular operate under tight resource limits [216,245]. A pragmatic sequence is therefore to begin with a managed-environment facility in which compound stress can be imposed with defined intensity and timing [51] to advance only lines that survive that stage to multi-location testing, and to increase the number of environments as the candidate set narrows. Genomic prediction can partially compensate for a limited number of tested environments by exploiting relationships among lines, although it does not remove the need for phenotypic anchors in the target environments [216]. Stability is ultimately an empirical property of a genotype within a defined population of environments: it cannot be established by mechanistic reasoning, nor inferred from a single site however favourably managed.

## 9. Translation, Governance and Equitable Deployment

Moving compound-stress-resilient edited wheat from research to the farmer’s field requires navigating heterogeneous regulatory landscapes [241]. The legal status of genome-edited crops varies widely: some jurisdictions treat transgene-free genome-edited plants as conventional, whereas others classify all genome-edited organisms as genetically modified organisms [242]. These differences in market regulation determine which editing options are feasible for each market [243]. Where applicable, transgene-free editing by RNP delivery or by segregation of the transgene can ease regulatory constraints, but requires editing that is attainable without stable transformation [249]. Freedom to operate is further constrained by intellectual property, including underlying CRISPR patents and trait patents, in both public-sector and commercial breeding [250]. Public-sector access to editing technologies and enabling germplasm is therefore key to meeting the needs of smallholder wheat producers in climate-exposed regions [251].

Technical success does not guarantee adoption [252]. A variety that is high-yielding and stable under compound stress will still fail if its grain quality is poor, if seed systems cannot deliver it, if it is poorly matched to local environments and farming practices, or if farmers and consumers do not prefer it [253]. Farmer participation is therefore necessary both in defining the target product profile and in evaluating edited lines [244]. Cultivars adapted to historical stress regimes may also be poorly matched to a climate that generates novel stress combinations [254,255]. Because future climate variables are uncertain, breeding for broad yield stability across variable environments is more robust than breeding for any single predicted scenario, and maintenance breeding and continued monitoring are essential for trait durability across genetic, pathogen and climate backgrounds [256].

## 10. Conclusions and Research Priorities

Wheat resilience to compound climate extremes will require context-dependent engineering of stress-integration networks rather than constitutive single-gene interventions [257]. The temporal structure of exposure—concurrent, sequential, recurrent or chronic with acute extremes—creates states that cannot be predicted from single-stress responses [34], and protection–productivity trade-offs demand tunable rather than constitutive solutions [103]. Because each homoeologue is present at three dosage levels, the hexaploid genome converts this requirement into a phenotypic gradient rather than a binary response [168].

The most promising interventions fall into two groups. Group 1, which can be tested experimentally in the near term, comprises guard-cell-specific, stress-inducible modulation of stomatal regulators, balancing water conservation against leaf cooling [76]; homoeologue-dosage tuning of signal-integration nodes such as ABA receptors and MAP kinases [148,152]; and promoter editing of source-sink regulators to maintain grain filling without compromising remobilization [161]. Group 2, which additionally requires multi-environment field validation, comprises developmental-stage-specific protection of meiosis, anthesis and grain filling through tissue-specific promoter editing, for which rice promoters provide only the conceptual basis [258] (C) and wheat elements remain to be validated. Evidential problems remain [259]: no wheat stress-memory mechanism has been causally established and field-tested [146], most targets rest on a single controlled-environment stress [219], and the dosage–phenotype relationship is unexplored in wheat [174,175].

Four falsifiable priorities follow. First, generate and field-test a full homoeologue-dosage series for at least three signal-integration genes in wheat, determining yield under concurrent, sequential and recurrent stress [260]. Second, causally test a wheat chromatin-modification memory mechanism by epigenome editing and determine whether the re-exposure response changes accordingly [132]. Third, validate guard-cell-specific wheat promoter elements that behave consistently across genetic backgrounds and test whether the stomatal closure rate can integrate water conservation with canopy cooling [75,76]. Fourth, determine whether the regulatory logic of stress-responsive promoter elements survives editing and recombination with different coding sequences [190]. Integrating temporal stress biology, trade-off characterisation, homoeologue-dosage logic and field-level yield-stability validation is a prerequisite for progress, not an optional refinement [21].

## Figures and Tables

**Figure 1:**
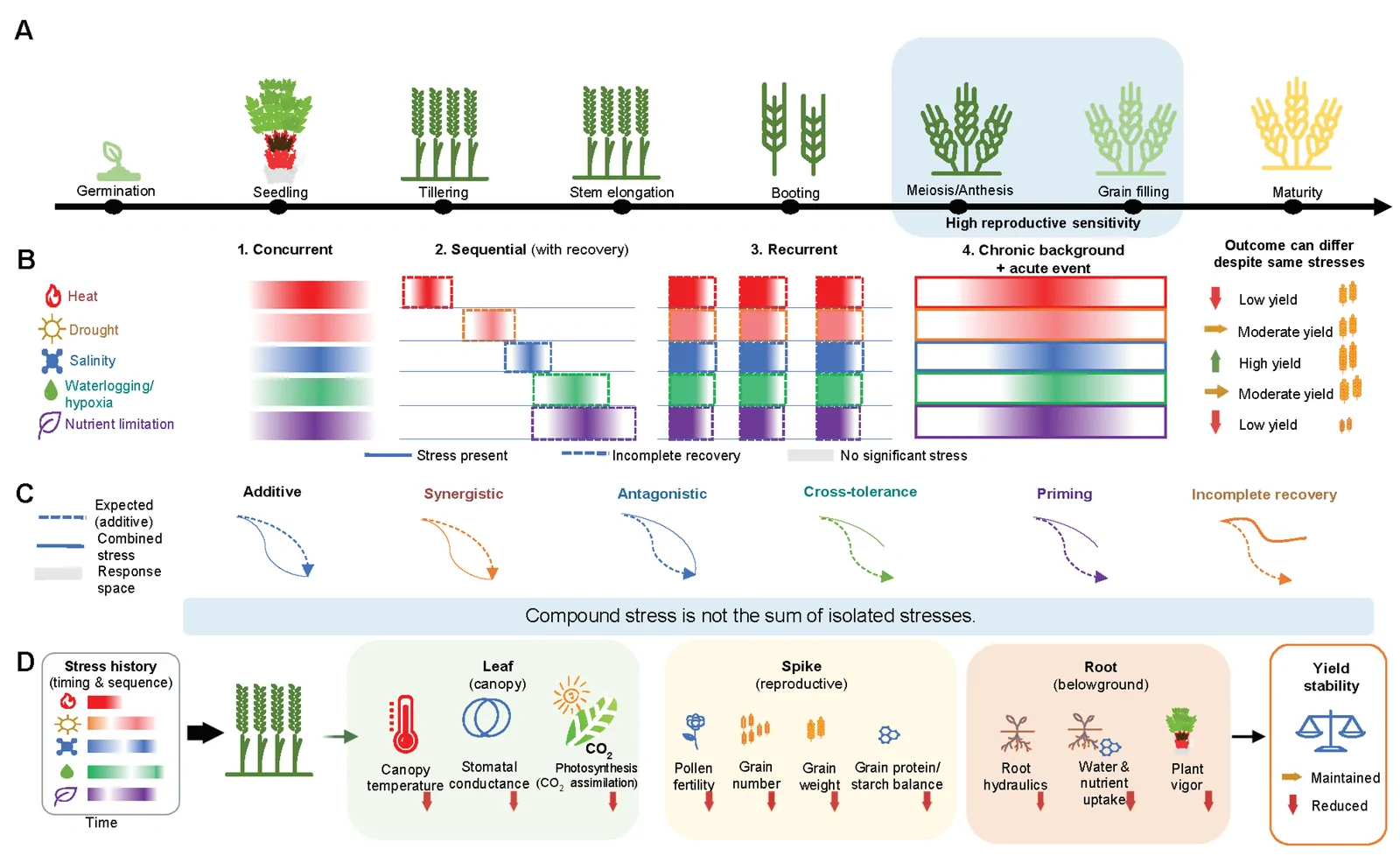
Temporal architecture of compound climate extremes across wheat development. (**A**) Developmental stages from germination to maturity, highlighting reproductive growth (meiosis, anthesis, grain filling) as the periods of greatest sensitivity. (**B**) Temporal stress configurations: concurrent, sequential, recurrent, and chronic background plus acute event, with inter-stress recovery intervals indicated; the same stress can have different consequences at different developmental stages. (**C**) Modes of interaction between combined stresses: additive, synergistic, antagonistic, cross-tolerance, priming and incomplete recovery; compound-stress phenotypes cannot be reconstructed from individual stress responses. (**D**) Agronomic implications linking stress history to yield-determining processes, from canopy temperature and stomatal conductance through pollen fertility and grain number to grain weight and yield stability. ABA, abscisic acid; ROS, reactive oxygen species. The figure is an original synthesis by the authors; supporting evidence is cited in the corresponding sections of the main text.

**Figure 2:**
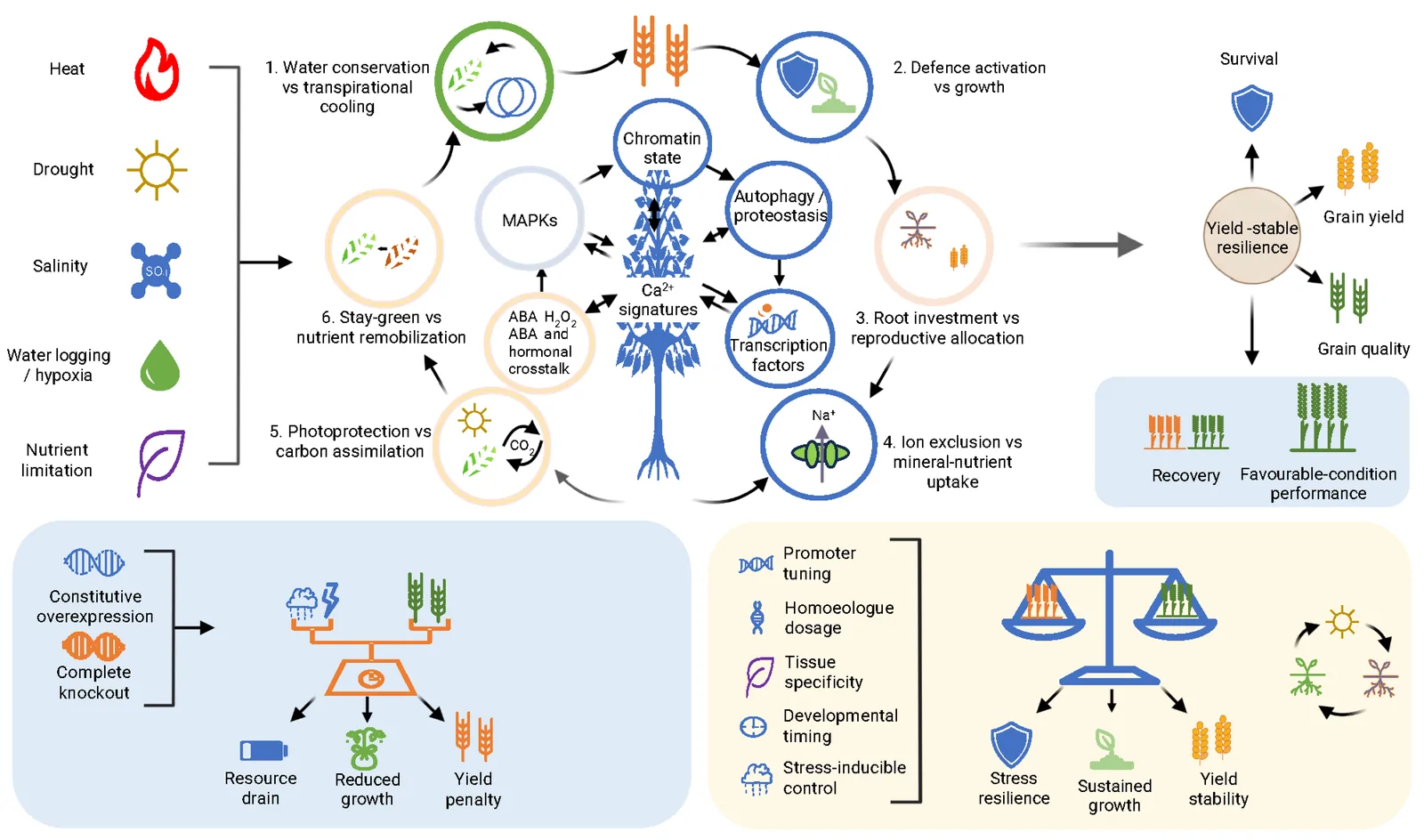
Molecular and physiological trade-offs governing yield-stable resilience. Six relationships between wheat plant functions are represented as balanced scales: (1) water conservation versus transpirational cooling; (2) defence activation versus growth; (3) root investment versus reproductive allocation; (4) ion exclusion versus mineral-nutrient uptake; (5) photoprotection versus carbon assimilation; and (6) stay-green versus nutrient remobilization. Central integrating components (calcium, ROS, ABA, MAPK, SnRK1–TOR, transcription factors and chromatin state) and the point at which environmental inputs converge are shown. The lower panel contrasts constitutive overexpression or knockout (unbalanced, potential yield loss) with promoter tuning, homoeologue dosage, tissue specificity and stress-inducible expression (balanced response). The figure is an original synthesis by the authors; supporting evidence is cited in the corresponding sections of the main text.

**Figure 3:**
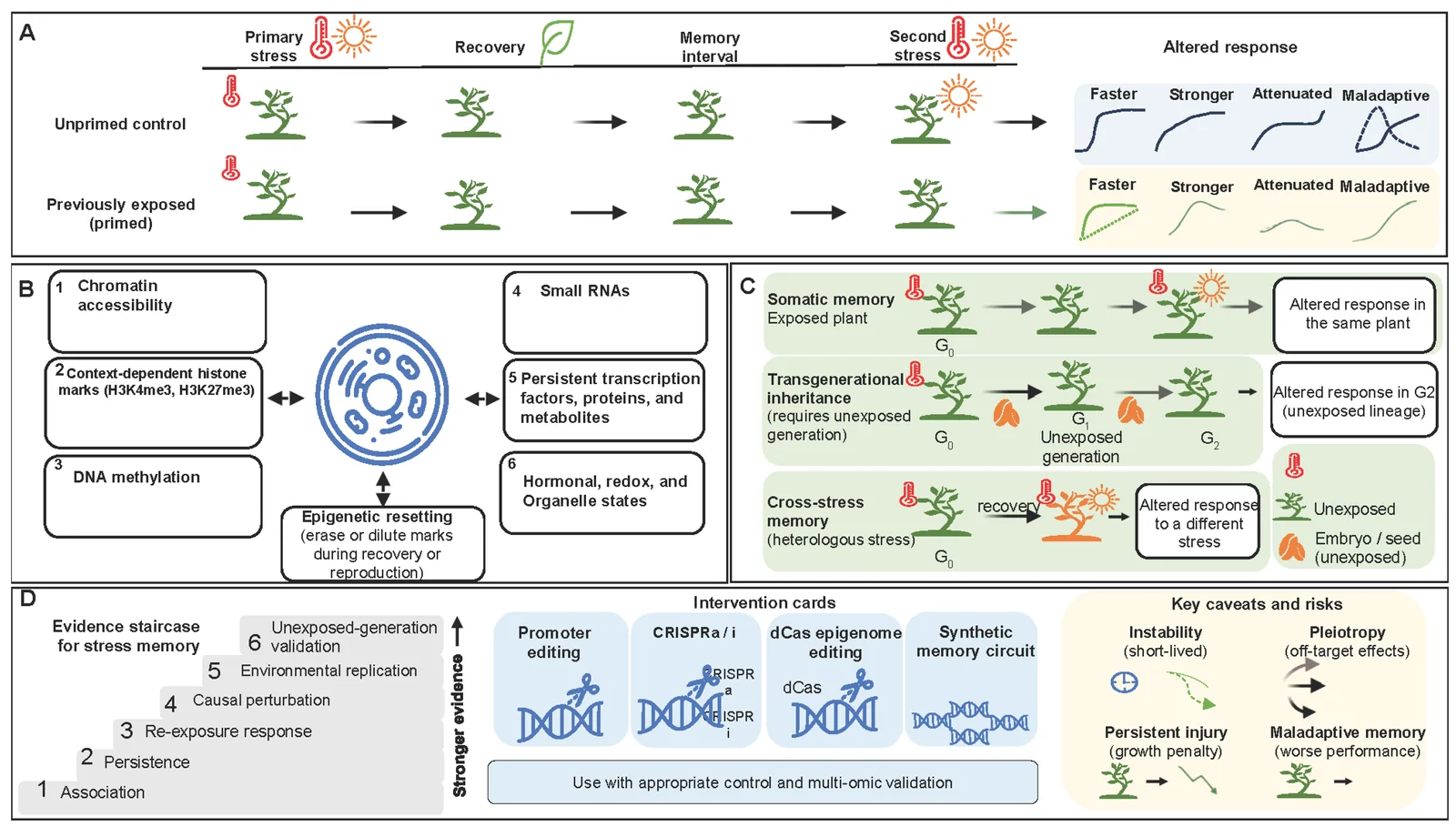
Mechanistic layers and evidential thresholds of plant stress memory. (**A**) Exposure timeline comprising first stress, recovery period, second stress and resulting response; second responses may be enhanced, dampened or maladaptive relative to unprimed plants. (**B**) Molecular memory layers: chromatin accessibility, histone modifications (H3K4me3 and H3K27me3 shown as examples), DNA methylation, small RNAs, persistent transcription factors and metabolites, and hormonal or redox states; dashed arrows denote uncertainty. (**C**) Biological duration, distinguishing somatic memory, intergenerational effects and transgenerational inheritance, and separating unexposed generations from directly exposed plants or embryos. (**D**) Evidence staircase: association, persistence, altered response after re-exposure, causal perturbation, environmental replication and validation in unexposed generations. Symbols mark candidate engineering interventions (promoter editing, CRISPRa/i, dCas9-based epigenome editing, synthetic circuits); warning symbols denote instability, pleiotropy and maladaptive memory. The figure is an original synthesis by the authors; supporting evidence is cited in the corresponding sections of the main text.

**Figure 4:**
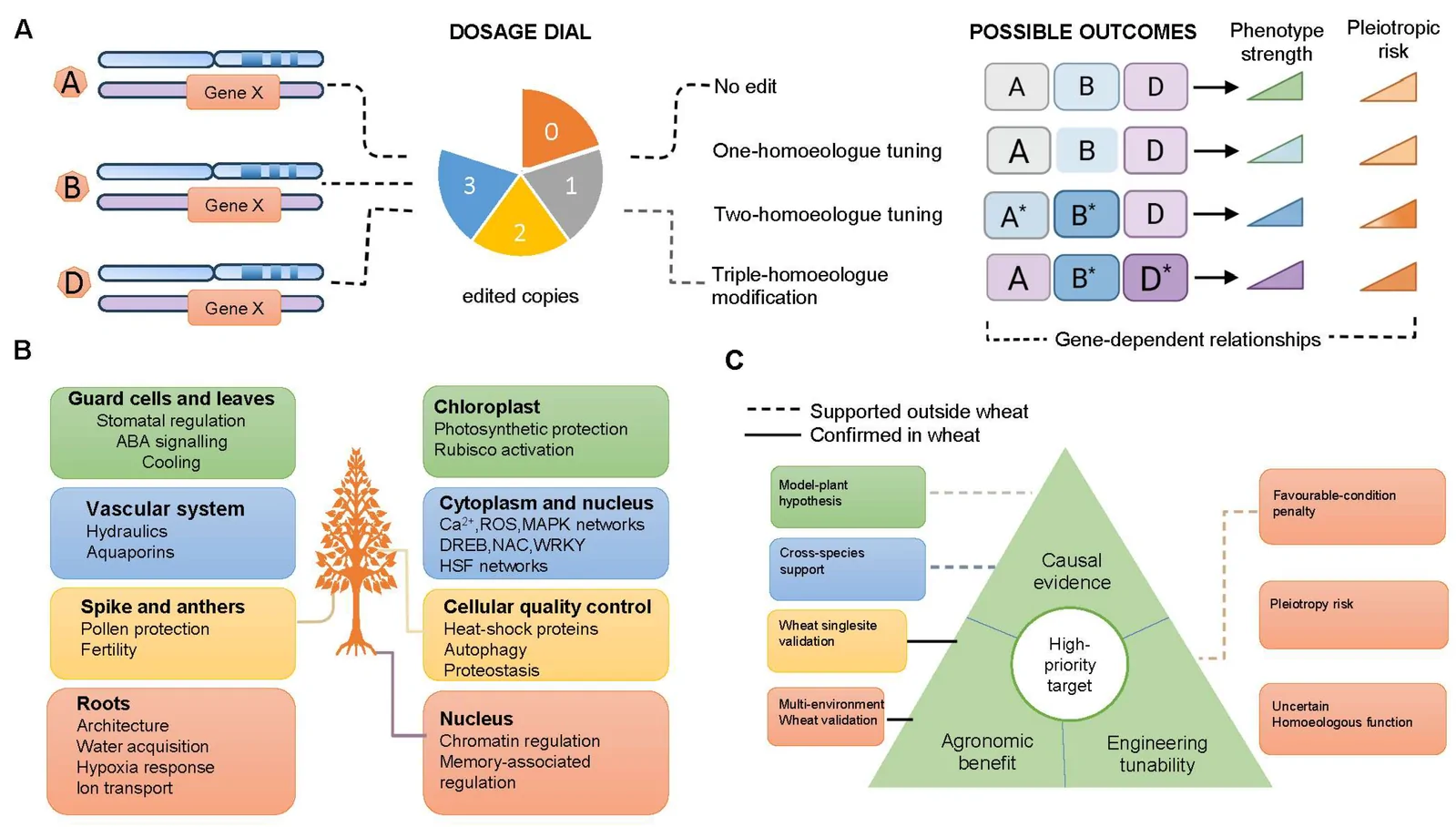
Target landscape and homoeologue-dosage logic for engineering hexaploid wheat. (**A**) Hexaploid dosage logic, showing the A, B and D chromosome ribbons and a representative homoeologue triad; a four-position dosage dial indicates that dosage, and therefore both phenotypic strength and pleiotropic risk, can be escalated stepwise. (**B**) Spatial target landscape: the guard cell and leaf, the vascular system, the root, the chloroplast, the cytoplasm and nucleus, cellular quality control, the spike and anther, the grain, and the nucleus. (**C**) Target prioritisation framework with causal evidence, agronomic benefit and engineering tunability at the vertices and high-priority targets in the central overlap; evidence-readiness badges range from model-plant hypothesis to multi-environment wheat validation. The figure is an original synthesis by the authors; supporting evidence is cited in the corresponding sections of the main text.

**Figure 5:**
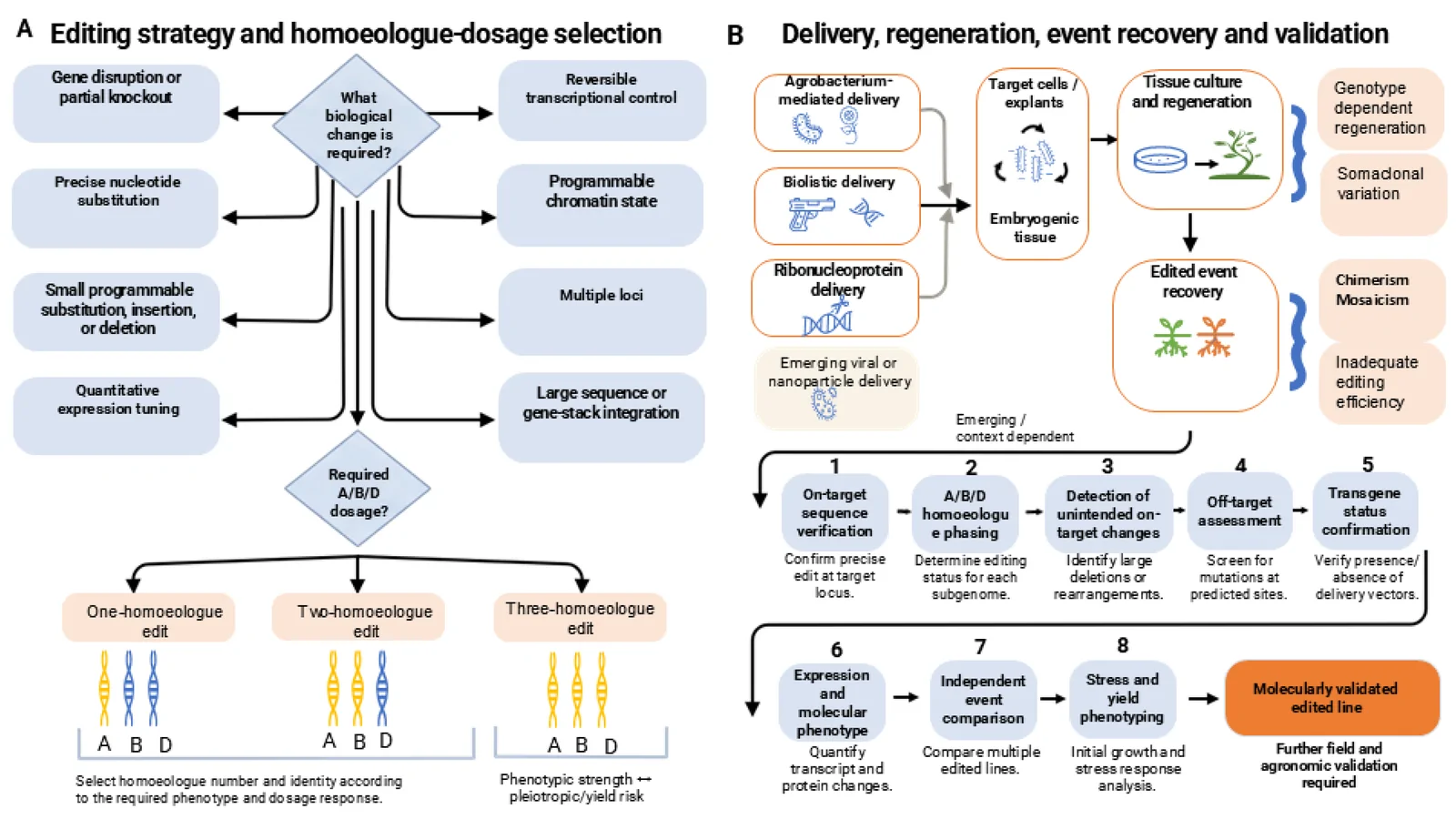
Decision framework for selecting precision-engineering strategies in wheat. (**A**) The first decision is the biological change to be introduced, with branches toward multiplex editing, insertion, chromatin-state editing, transcriptional control with CRISPRa and CRISPRi, quantitative promoter tuning, base editing and targeted disruption with Cas9 or Cas12a; each branch requires an explicit determination of dosage (one, two or three of the A, B or D homoeologues), and constraint tags indicate PAM availability, editing window, bystander changes and efficiency. (**B**) Delivery to a validated line: input routes, tissue culture and regeneration, and the quality-control pipeline, with failure branches for genotype-dependent regeneration, somaclonal variation and insufficient editing efficiency. The figure is a conceptual synthesis by the authors; supporting evidence—including evidence from other cereals (C) and model species (M)—is cited in Section 7.1, Section 7.2, Section 7.3 and Section 7.4 [167,168,169,170,171,172,173,174,177,179,181,183,184,190,192,193,194,195,196,197,198,199,202,203,204,206,207,208,210,211,212,213].

**Table 1:** Molecular and agronomic trade-offs in engineering wheat resilience.

Trait or Intervention	Intended Benefit	Opposing Physiological Cost	Stress Context	Consequence Under Favourable Conditions	Effect on Yield or Grain Quality	Strategy for Reducing Trade-Off	Evidence Level	Supporting References
Enhanced stomatal closure (ABA hypersensitivity)	Water conservation under drought	Reduced transpirational cooling, elevated leaf temperature	Detrimental under combined heat + drought	Reduced photosynthesis, lower biomass	Yield penalty under heat or favourable conditions	Guard-cell-specific modulation; heat-responsive override of ABA signalling	B/C	[55,56,57,73,74,75]
Constitutive antioxidant overexpression	Protection from oxidative damage	Reduced ROS signalling for acclimation; metabolic cost	Beneficial under acute stress; detrimental under moderate stress	Growth reduction from unnecessary protein production	Yield penalty under moderate or favourable conditions	Stress-inducible or recovery-tuned expression	C/D	[81,108]
Increased root biomass or depth	Improved water and nutrient acquisition	Carbon diversion from reproductive sinks	Beneficial under terminal drought; neutral or negative under waterlogging	Reduced harvest index, lower grain number	Yield benefit under drought; penalty under favourable conditions	Developmental-stage-specific root growth promotion	B/C	[83,84]
Sodium exclusion (HKT1;5 enhancement)	Reduced leaf sodium toxicity under salinity	Impaired potassium uptake under low K^+^ availability	Beneficial under salinity; detrimental under combined salinity + low K^+^	None under adequate K^+^; penalty under K^+^ limitation	Yield benefit under salinity; potential penalty under nutrient limitation	Root-specific expression; potassium-status-responsive regulation	B	[36,37,88,89]
Stay-green/delayed senescence	Extended grain-filling duration	Delayed nitrogen and carbon remobilization	Beneficial under moderate terminal stress; detrimental under severe stress	Reduced grain protein content	Increased grain weight; decreased protein concentration	Senescence timing matched to stress severity; inducible senescence acceleration	B/C	[90,91,93,95]
Constitutive DREB/NAC overexpression	Enhanced stress survival	Sustained growth repression via DELLA and ABA pathways	Beneficial under severe stress only	Severe growth reduction, delayed development	Yield penalty under well-watered conditions; magnitude not established in field-grown wheat	Stress-inducible promoters; tissue-specific expression	B/C	[12,13,98,99]
Osmolyte accumulation (proline, glycine betaine)	Osmotic adjustment and protein stabilisation	High energetic cost of biosynthesis	Beneficial under severe osmotic stress	Energetic cost reduces growth under favourable conditions	Yield benefit under severe drought; penalty under moderate or favourable	Stress-inducible synthesis; rapid turnover engineering	C/D	[100]
Enhanced heat-shock protein expression	Proteostasis maintenance under heat	ATP consumption; potential interference with protein turnover	Beneficial under acute heat; unclear under combined stress	Possible growth penalty from constitutive ATP consumption	Unknown in wheat field trials	Heat-inducible expression; recovery-timed downregulation	D/E	[109,110]
Reduced stomatal density	Improved water-use efficiency	Reduced CO_2_ uptake, potential heat buildup	Beneficial under drought; detrimental under high radiation	Reduced photosynthetic capacity	Yield reduction under favourable; potential benefit under drought	Developmental-stage-specific stomatal density control	B/C	[76,111]
Enhanced root cortical aerenchyma	Improved waterlogging tolerance	Reduced water transport under subsequent drought	Beneficial under waterlogging; detrimental if drought follows	None under waterlogging; penalty under drought	Variable depending on seasonal water pattern	Inducible aerenchyma formation only under hypoxia	D	[85]
Constitutive ABA accumulation	Pre-emptive stress protection	Growth inhibition; stomatal closure limiting photosynthesis	Generally detrimental except under severe chronic stress	Severe growth reduction	Major yield penalty	Stress-inducible rather than constitutive ABA elevation	B/C	[12,104]
Enhanced autophagy	Improved nutrient remobilization under stress	Potential excessive degradation under moderate stress	Beneficial under severe stress; detrimental under recovery	Premature senescence risk	Context-dependent	Stress-responsive autophagy with recovery-phase downregulation	C/D	[96]

Notes: Evidence levels are defined in Supplementary Table S1. ABA, abscisic acid; ROS, reactive oxygen species.

**Table 2:** Evidence-gated validation pathway for yield-stable edited wheat.

Development Gate	Minimum Experimental Design	Essential Controls	Measurements	Pass Criterion	Failure Warning	Reporting Requirement	Next Decision	Supporting References
Gate 1: Target selection	Bioinformatic and literature evidence for target in wheat stress response	N/A at selection stage	Literature evidence level; homoeologue annotation; expression data	Level B/C wheat evidence for target function; plausible engineering strategy identified	Level D/E only; no wheat expression data; unknown homoeologue function	Full evidence table with homoeologue information	Proceed to editing design if pass; generate wheat evidence if fail	[6,10,31]
Gate 2: Molecular validation	Edited T_0_/T_1_ plants with sequencing confirmation	Wild-type or null-segregant isogenic control	Sanger or NGS of targeted homoeologues; off-target screening; expression analysis; transgene status	Correct edit in intended homoeologue(s) without unintended rearrangements; off-targets absent or benign; defined expression change	Mosaic or chimeric plants; large deletions; off-target edits in stress-responsive genes; no expression change	Edit sequence, homoeologue phasing, off-target report, expression data	Proceed to controlled stress test if pass; redesign if fail	[211,212,236]
Gate 3: Controlled-environment stress test	Single stress, concurrent stress, sequential stress treatments at relevant developmental stage; recovery measurement	Isogenic null control; multiple independent edited events; unstressed treatment	Canopy temperature; gas exchange; chlorophyll fluorescence; biomass; yield components; recovery growth	Stress protection or recovery benefit in ≥2 independent events without >10% penalty under favourable conditions	No benefit over control; benefit in one event only; >15% favourable-condition penalty	All physiological and yield data for all events and controls	Proceed to managed-environment testing if pass; characterise mechanism if marginal; abandon if fail	[49,219,237]
Gate 4: Recovery and recurrent stress test	Stress treatment with defined recovery interval and stress re-exposure	Isogenic control; single-stress without recovery component	Recovery growth rate; re-exposure response; yield components after full cycle	Recovery benefit maintained; no maladaptive response to re-exposure	Incomplete recovery; worse re-exposure response than naïve control	Recovery time-course data; re-exposure physiology	Proceed to field testing if pass	[38,46,120]
Gate 5: Managed-environment field test	Rainout shelter or managed stress field with single and compound stress	Isogenic control; multiple events; locally adapted background	Canopy temperature; soil moisture; gas exchange; root traits where feasible; yield; grain quality; harvest index	Yield benefit under stress without reduction under favourable conditions; grain quality maintained	Yield penalty in any treatment; grain quality degradation	Full environmental characterisation; yield and quality data per treatment	Proceed to multi-location field trial if pass	[51,219]
Gate 6: Multi-location field trial	≥3 locations; ≥2 seasons; target population of environments	Isogenic control in each environment; local check cultivars	Yield; yield stability statistics; grain quality; phenology; disease/insect notes	Significant yield stability benefit; no environment with unacceptable performance; quality meets market standards	Genotype × environment interaction favouring control in any environment; quality failure	Individual environment data; combined stability analysis; quality profiles	Proceed to regulatory and deployment if pass	[222,223,230,231,234]
Gate 7: Favourable-environment validation	Well-watered, optimal nutrient, standard density trials in ≥2 locations	Isogenic control; elite check cultivars	Yield; harvest index; grain quality; phenology	Yield and quality statistically equivalent to or better than isogenic control	Yield penalty exceeding experimental error; delayed maturity; quality defect	Full agronomic data under optimal management	Proceed to deployment if pass; backcross and re-select if marginal	[12,98,99,107]
Gate 8: Grain quality assessment	Replicated grain samples from all field environments	Isogenic control; market-standard cultivars	Protein content; starch properties; dough rheology; micronutrients where relevant	All quality parameters within acceptable range for target market class	Any quality parameter outside market specification	Complete quality profile per environment	Proceed if pass; select within edited population if variation exists; abandon if consistent failure	[91,95]
Gate 9: Regulatory and biosafety characterisation	Molecular characterisation per relevant jurisdiction requirements	N/A	Event-specific detection method; compositional analysis; allergenicity where required	Meets jurisdiction-specific regulatory requirements for genome-edited crops	Failure to meet any required regulatory standard	Complete regulatory dossier	Proceed to breeding integration if pass	[241,242,243]
Gate 10: Breeding integration and deployment	Introgression into target cultivars; participatory evaluation	Original edited line; recipient cultivars	Agronomic performance after backcrossing; farmer acceptance; seed system compatibility	Trait maintained through backcrossing; farmer-preferred characteristics; seed production feasible	Trait loss during backcrossing; farmer rejection; seed system incompatibility	Breeding population performance; participatory evaluation results	Release decision; post-release monitoring	[205,215,244]

Notes: Evidence levels are defined in Supplementary Table S1. NGS, next-generation sequencing.

## Data Availability

No new data were created or analysed in this study. Data sharing is not applicable to this article. All material discussed is available in the cited published literature.

## Supplementary Materials

The following supporting information can be downloaded at: https://www.mdpi.com/article/10.3390/plants15192936/s1, Table S1: Operational terminology and evidential standards; Table S2: Climate-relevant compound stresses and their consequences in wheat; Table S3: Stress-memory mechanisms and their engineering readiness; Table S4: Target-tool matrix for precision engineering of compound-stress resilience; Table S5: Search strategy, screening flow and evidence-classification criteria; Table S6: Operational terms and minimum design requirements; Table S7: Species-of-origin markers and inference rules.

## Abbreviations

The following abbreviations are used in this manuscript:

ABA, abscisic acid; ABRE, ABA-responsive element; AMMI, additive main effects and multiplicative interaction; CBL, calcineurin B-like protein; CDPK, calcium-dependent protein kinase; CIPK, CBL-interacting protein kinase; CRISPR, clustered regularly interspaced short palindromic repeats; CRISPRa, CRISPR activation; CRISPRi, CRISPR interference; dCas9, catalytically dead Cas9; DRE, dehydration-responsive element; DREB, dehydration-responsive element-binding protein; G × E, genotype × environment interaction; G × E × M, genotype × environment × management interaction; GGE, genotype plus genotype-by-environment interaction; GRF-GIF, GROWTH-REGULATING FACTOR-GRF-INTERACTING FACTOR; gRNA, guide RNA; HAT, histone acetyltransferase; HDAC, histone deacetylase; HKT, high-affinity potassium transporter; HSF, heat-shock factor; HSP, heat-shock protein; MADS-box, MCM1-AGAMOUS-DEFICIENS-SRF box; MAPK, mitogen-activated protein kinase; MLO, MILDEW RESISTANCE LOCUS O; NAC, NAM-ATAF1/2-CUC2 transcription factor family; NHX, Na+/H+ exchanger; PAM, protospacer-adjacent motif; PIP, plasma membrane intrinsic protein; PP2C, type 2C protein phosphatase; PRC2, Polycomb repressive complex 2; PYR/PYL/RCAR, pyrabactin resistance/PYR1-like/regulatory component of ABA receptor; QTL, quantitative trait locus; RNP, ribonucleoprotein; ROS, reactive oxygen species; siRNA, small interfering RNA; SLAC1, slow anion channel-associated 1; SnRK1/SnRK2, sucrose non-fermenting-1-related protein kinase 1/2; SOS1, salt overly sensitive 1; SUT, sucrose transporter; SWEET, sugars will eventually be exported transporter; T6P, trehalose 6-phosphate; TIP, tonoplast intrinsic protein; TOR, target of rapamycin; TPE, target population of environments; TPP, trehalose 6-phosphate phosphatase; TPS, trehalose 6-phosphate synthase; VPD, vapour pressure deficit; WRKY, WRKY-domain transcription factor family; WUE, water-use efficiency.
