# Supplementary material for: Engineering Wheat for Compound Climate Extremes: Molecular Trade-Offs, Stress Memory, and Precision Genome Editing for Yield-Stable Resilience
Source: Plants (Basel). 2026 Sep 25;15(19):2936. doi: 10.3390/plants15192936 (PMC13666858; doi:10.3390/plants15192936)
Supplement: Supplementary file 1 [file plants-15-02936-s001.zip › plants-4537117-supplementary.pdf]

Supplementary Materials

Engineering Wheat for Compound Climate Extremes: Molecular Trade-Offs, Stress Memory, and Precision Genome Editing for Yield-Stable Resilience

Xin Song[1*], Xixiong Hong[1], Huaiyuan Wu[1], Peipei Su[1] and Minshan Sun[2*]

This file contains one supplemental figure and seven supplemental tables. All items are cited in the main text.

Contents

• Table S1. Operational terminology and evidential standards.

Table S1 - cited in Sections 2, 5.3 and 6.6, and in Tables 1 and 2

Table S1 - cited in Sections 2, 5.3 and 6.6, and in Tables 1 and 2• Table S6. Operational terms and the minimum experimental design required to support them.

Table S1 - cited in Sections 2, 5.3 and 6.6, and in Tables 1 and 2• Table S6. Operational terms and the minimum experimental design required to support them. Table S6 - read with Section 2.2

Table S1 - cited in Sections 2, 5.3 and 6.6, and in Tables 1 and 2• Table S6. Operational terms and the minimum experimental design required to support them. Table S6 - read with Section 2.2• Table S7. Species-of-origin markers and the claim strength each permits.

Table S1 - cited in Sections 2, 5.3 and 6.6, and in Tables 1 and 2• Table S6. Operational terms and the minimum experimental design required to support them.Table S6 - read with Section 2.2• Table S7. Species-of-origin markers and the claim strength each permits.Table S7 - read with Section 2.3

• Table S2. Climate-relevant compound stresses and their consequences in wheat.

Table S2 - cited in Section 3.3

• Table S3. Stress-memory mechanisms and their engineering readiness.

Table S3 - cited in Section 5.3

• Table S4. Target-tool matrix for precision engineering of compound-stress resilience.

Table S4 - cited in Section 6.6

Table S4 - cited in Section 6.6• Table S5. Search strategy, screening flow and evidence-classification criteria.

Table S4 - cited in Section 6.6• Table S5. Search strategy, screening flow and evidence-classification criteria. Table S5 - read with Section 2.1

Table S1. Operational terminology and evidential standards.

Term	Operational Definition	Temporal or Generational Scale	Minimum Experimental Design	Evidence Required	Common Misuse	Engineering Implication
Compound stress	Simultaneous exposure to two or more abiotic stressors	Within single stress event	Single vs combined stress treatments with factorial design	Non-additive molecular or physiological response	Applied to any multifactorial experiment regardless of interaction	Engineering must address stress interactions, not single-stress mechanisms
Concurrent stress	Two or more stressors applied simultaneously	Overlapping exposure periods	Concurrent treatment with matched single-stress controls	Demonstration of concurrent-specific responses distinct from sequential	Conflated with sequential stress	Demands integration-node rather than pathway-specific engineering
Sequential stress	Temporally separated stressors where order may matter	Hours to weeks between events	Defined sequence with recovery interval; reversed-order control	Order-dependent outcomes attributable to first stress altering second response	Described without recovery-interval documentation	First stress response may be engineerable to modify second stress outcome
Recurrent stress	Repeated exposure to same stressor(s) with intervening recovery	Multiple cycles within one generation	Repeated stress-recovery cycles; single-exposure control	Altered response across cycles consistent with priming or exhaustion	Single repeated measurement treated as recurrent exposure	Memory mechanisms become relevant engineering targets
Acclimation	Gradual physiological adjustment to sustained stress	Hours to days	Gradual vs abrupt stress imposition	Improved function under gradual vs abrupt stress	Confused with genetic adaptation or priming	Engineering should consider acclimation capacity, not just acute responses
Priming	Prior exposure causing modified response to subsequent stress	Within-generation memory	Priming treatment, recovery, challenge stress; unprimed challenge control	Altered response attributable to prior exposure, not residual damage	Any prior stress exposure called priming	Priming mechanisms engineerable without constitutive activation cost
Cross-tolerance	Exposure to one stress enhancing tolerance to different stress	Within-generation; may persist after first stress relief	Stress A, recovery, stress B design; single-stress controls	Enhanced tolerance to stress B not explained by stress A defence overlap	Assumed from overlapping gene lists	Enables broad-spectrum engineering from narrower priming interventions
Resilience	Capacity to maintain function and yield under stress and recover upon stress relief	Within-generation; multi-environment assessment	Stress imposition, recovery measurement, yield assessment under variable conditions	Maintained yield and grain quality with recovery capacity across environments	Equated with survival or single-stress tolerance	Engineering target is yield stability, not stress survival
Somatic memory	Persistent biological state within a single plant generation	Within one generation	Stress, recovery interval, re-exposure; naive control	Persistent molecular state and altered re-exposure response	Any transcriptional change after stress called memory	Memory mechanism must be causally validated before engineering
Intergenerational effect	Phenotype in offspring influenced by parental environment	One generation; parental exposure to offspring phenotype	Parental stress treatment; offspring tested without stress; reciprocal crosses	Offspring phenotype difference not explained by seed provisioning	Parental effects assumed transgenerational	May be exploitable through parental treatment but not stable inheritance
Transgenerational memory	Heritable stress effect persisting in unexposed generations	Two or more unexposed generations	Stress, multiple unexposed generations with environmental controls; reciprocal crosses	Persistent phenotype in G2+ unexposed without environmental carryover	Claimed from single-generation offspring effects	Would enable heritable resilience but remains largely unvalidated

Operational definitions of the temporal stress and memory terms used throughout the review, with the minimum experimental design and evidence required to support each claim.

Table S2. Climate-relevant compound stresses and their consequences in wheat.

Stress Architecture	Developmental Stage	Key Molecular or Physiological Interaction	Response Type	Yield or Grain Quality Consequence	Wheat Evidence Strength	Major Experimental Limitation	Priority Research Gap
Concurrent heat + drought	Anthesis	ABA-mediated stomatal closure exacerbates heat damage to pollen	Synergistic	Floret sterility, grain number reduction > additive prediction	Strong (Level B)	Field studies rarely capture coincident peak stress	Guard-cell-specific engineering to decouple water saving from cooling
Concurrent heat + drought	Grain filling	Reduced photosynthesis with accelerated senescence	Synergistic	Grain weight reduction, altered protein/starch ratio	Strong (Level B)	Genotype × stress timing interactions poorly resolved	Source–sink engineering for maintained remobilization
Sequential drought then heat	Grain filling	Prior drought depletes reserves for heat response	History-dependent	Greater grain weight loss than drought or heat alone	Moderate (Level C)	Recovery interval between stresses rarely standardized	Recovery-promoting interventions between sequential stresses
Sequential waterlogging then drought	Tillering to stem elongation	Hypoxia damages root function before terminal drought	History-dependent	Reduced water extraction, premature senescence	Moderate (Level C)	Root phenotype recovery rarely quantified	Root-system engineering for hypoxia–drought resilience
Recurrent heatwaves	Reproductive development	Progressive pollen viability loss, antioxidant depletion	Cumulative	Greater sterility than single heat event of equal duration	Moderate (Level C)	Frequency and intensity interactions understudied	Heat-priming interventions between heatwave events
Chronic drought + acute heatwave	Grain filling	Depleted soil water prevents transpirational cooling during heat	Synergistic	Canopy temperature lethal threshold exceeded	Strong (Level B)	Chronic stress intensity gradients rarely controlled	Threshold-based stress-responsive engineering
Concurrent salinity + drought	Vegetative to reproductive	Osmotic stress compounded by ionic toxicity	Additive to synergistic	Biomass and yield reduction exceeding single stresses	Moderate (Level C)	Combined treatments rarely applied at field-relevant levels	Ion-transporter engineering under combined osmotic–ionic stress
Salinity + heat	Anthesis to grain filling	Sodium accumulation exacerbates heat-induced oxidative damage	Synergistic	Spikelet sterility, grain sodium accumulation	Limited (Level C/D)	Very few wheat studies with controlled combined treatment	Ion-homeostasis engineering under combined stress
Nutrient limitation + heat	Grain filling	Nitrogen remobilization disrupted under heat	Antagonistic to additive	Grain protein reduced more than starch	Moderate (Level B)	Nutrient × heat interactions genotype-dependent	Nutrient-use efficiency under combined stress
Waterlogging followed by rapid soil drying	Stem elongation to booting	Root cortical aerenchyma reduces water transport after drainage	History-dependent	Impaired water uptake during subsequent drought	Limited (Level C)	Soil moisture dynamics after waterlogging rarely monitored	Root anatomical engineering for dual-stress function
Recurrent drought cycles	Vegetative to reproductive	Priming of antioxidant and osmotic adjustment	Cross-tolerant potential	Variable; priming may improve terminal stress tolerance	Limited (Level C)	Priming vs cumulative damage difficult to distinguish	Memory-based engineering for drought recurrence
Combined heat + drought + high VPD	Anthesis	Extreme evaporative demand with limited water supply	Synergistic	Complete reproductive failure in sensitive genotypes	Moderate (Level B)	VPD rarely controlled independently of temperature	Stomatal and cuticular engineering for high VPD tolerance

ABA, abscisic acid; VPD, vapour pressure deficit.

Table S3. Stress-memory mechanisms and their engineering readiness.

Memory Layer	Proposed Mechanism	Persistence Period	Wheat Evidence	Supporting Cereal or Model Evidence	Causality Status	Potential Engineering Approach	Stability or Pleiotropy Concern
H3K4me3 hypermethylation at primed loci	Sustained chromatin accessibility at stress-responsive genes	Days to weeks (somatic)	Correlative only (no causal demonstration; not Level C)	Causal in Arabidopsis (Level E)	Correlation only in wheat	dCas9–histone methyltransferase fusion targeted to stress loci	Genome-wide effects if modifiers broadly expressed; specificity challenge
H3K27me3 dynamics at developmental genes	Altered Polycomb repression affecting growth–defence balance	Days to weeks (somatic)	Correlative only (no causal demonstration; not Level C)	Causal in Arabidopsis (Level E)	Correlation only in wheat	dCas9–PRC2 component fusion for locus-specific H3K27me3 deposition	Developmental pleiotropy risk; resetting during reproduction
DNA methylation at stress-responsive transposons and promoters	Altered transcriptional responsiveness upon re-exposure	Weeks to months; limited transgenerational	Correlative only (no causal demonstration; not Level C)	Mixed causal evidence in Arabidopsis (Level E)	Correlation only; evidence against stable inheritance in wheat	dCas9–DNA methyltransferase fusion for targeted methylation	Passive and active demethylation during reproduction; instability across environments
Small RNA-mediated systemic memory	Mobile small RNAs propagating primed state systemically	Days to weeks (somatic)	Limited (Level D/E)	Causal in Arabidopsis for specific siRNAs (Level E)	Correlation only in cereals	Synthetic small RNA circuits; mobile small RNA engineering	Delivery and stability of engineered small RNAs unvalidated in wheat
Persistent heat-shock factor (HSF) activity	Autoregulatory HSF loops maintaining HSP expression	Hours to days (somatic)	Limited (Level D)	Demonstrated in Arabidopsis (Level E)	Partial causal evidence in model plants	Promoter editing for sustained HSF autoregulation	Extended HSP expression consumes ATP; recovery-phase silencing needed
Sustained ABA elevation and signalling	Prolonged ABA biosynthesis and reduced catabolism after stress	Days (somatic)	Correlative only (no causal demonstration; not Level C)	Causal in Arabidopsis (Level E)	Correlation only	Promoter editing of ABA catabolic genes (CYP707A) for controlled ABA persistence	Constitutive growth repression if ABA remains elevated
Osmolyte and metabolite carryover	Maintained compatible solute concentrations after stress relief	Days to weeks depending on turnover	Correlative only (no causal demonstration; not Level C)	Demonstrated in multiple species	Correlation only; causal role in memory unclear	Engineering osmolyte turnover rates	Energetic cost of maintained osmolyte pools
Redox-state memory	Sustained alterations in ROS-scavenging enzyme activities	Days (somatic)	Correlative only (no causal demonstration; not Level C)	Causal evidence limited	Correlation only; ROS-scavenging vs signalling roles confounded	Promoter editing of antioxidant genes for sustained induction	Impaired ROS signalling if scavenging is excessive
Synthetic toggle-switch memory circuits	Engineered positive feedback loops maintaining gene expression state	Designed persistence	None in wheat (Level E)	Proof of concept in yeast, mammalian cells (Level E)	None in plants	Synthetic transcription factor circuits with positive feedback	Transgene complexity, regulatory issues, stability in field
Heat-primed pollen thermotolerance	Maintained HSP and compatible solute levels in developing pollen	Days within reproductive phase (somatic)	Limited (Level D)	Rice evidence (Level D)	Correlation only	Stage-specific pollen HSP engineering	Pollen-specific expression technically challenging in wheat

HSF, heat-shock factor; HSP, heat-shock protein; PRC2, Polycomb repressive complex 2; siRNA, small interfering RNA.

Table S4. Target-tool matrix for precision engineering of compound-stress resilience.

Biological Module	Representative Target or Target Class	Intended Phenotype	Relevant Compound-Stress Scenario	Preferred Editing Strategy	A/B/D Homoeologue Strategy	Principal Trade-Off or Risk	Current Readiness Level
ABA perception and signalling	PYL/RCAR ABA receptors	Adjusted ABA sensitivity; balanced water conservation and cooling	Concurrent heat + drought	Promoter editing for guard-cell-enriched expression; base editing of ligand-binding domain	1–2 homoeologues edited to avoid complete loss	Excessive ABA sensitivity exacerbates heat damage through stomatal closure	Level C: PYL function known in wheat, guard-cell engineering not demonstrated
ABA signalling negative regulation	PP2C phosphatases (ABI1, ABI2 homologues)	Reduced PP2C activity for enhanced drought response	Sequential drought then heat	Promoter editing for stress-inducible downregulation	Partial dosage across homoeologues	Constitutive PP2C loss causes extreme ABA hypersensitivity and growth arrest	Level C/D: Demonstrated in Arabidopsis; wheat homoeologues identified
Calcium signalling	CDPKs, CBL–CIPK modules	Altered stress-prioritization signalling under combined stress	Concurrent heat + drought; salinity + heat	Base editing of calcium-binding or activation domains	Homoeologue-specific editing based on expression dominance	Disrupted calcium signalling affects development and pathogen responses	Level D/E: Wheat CDPK functions partially characterized
MAPK signalling integration	MPK3, MPK6 homologues	Tuned MAPK dynamics for compound-stress-specific outputs	Multiple compound stresses	Promoter editing; phosphorylation-site base editing	One homoeologue edited to reduce rather than eliminate activity	MAPKs regulate diverse processes including growth and immunity	Level D: MAPK stress roles known in cereals, engineering not demonstrated
Stomatal development	SPCH, MUTE, FAMA	Reduced or redistributed stomatal density	Chronic drought + heatwaves	Promoter editing for epidermis-specific expression modulation	All three homoeologues likely required	Reduced stomatal density improves WUE but limits CO_2 uptake	Level C/D: Wheat stomatal development genes identified; editing not reported
Guard-cell ion transport	SLAC1, GORK, KAT1/2	Adjusted stomatal aperture dynamics	Concurrent heat + drought; high VPD	Base editing of regulatory domains; guard-cell-specific promoter editing	Homoeologue expression profiling needed	Altered stomatal kinetics affect both water loss and CO_2 uptake	Level D/E: Wheat guard-cell channel function inferred from other species
Aquaporin regulation	PIP1, PIP2, TIP subfamilies	Maintained root hydraulic conductivity under combined stress	Waterlogging followed by drought; salinity + drought	Promoter editing for stress-responsive expression maintenance	Homoeologue-specific; functional divergence among isoforms	Aquaporin overexpression can increase water loss under drought	Level C: Wheat aquaporin expression characterized; editing not reported
Sodium exclusion	HKT1;4, HKT1;5	Enhanced sodium exclusion without potassium compromise	Salinity + drought; salinity + heat	Promoter editing for root-stele-specific expression; allelic replacement with natural variants	4D homoeologue (HKT1;5) primary target; A and B copies may require different strategy	Sodium exclusion can impair potassium uptake under low K^+	Level B: Field-validated natural alleles; editing for enhanced function not reported
Vacuolar sodium sequestration	NHX1, NHX2	Compartmentalized sodium without disrupting vacuolar K^+ and Ca^{2+}	Salinity + heat	Promoter editing for stress-inducible expression; base editing for altered cation selectivity	Multiple homoeologues likely required	Vacuolar overloading may disrupt turgor regulation	Level C/D: NHX function known in cereals; selectivity engineering not demonstrated
Antioxidant regulation	ROS-scavenging enzymes (APX, CAT, SOD, GR)	Enhanced ROS detoxification under compound stress without signalling disruption	Heat + drought; salinity + heat	Stress-inducible promoter engineering; compartment-specific targeting	Partial homoeologue editing to avoid complete loss	Excessive scavenging impairs ROS signalling required for acclimation	Level C/D: Individual enzyme overexpression tested; stress-inducible systems not developed
Heat-shock response	HSF transcription factors (HSFA2, HSFA6)	Sustained proteostasis protection under recurrent heat	Recurrent heatwaves; heat + drought	Promoter editing for heat-inducible expression with recovery-phase	Homoeologue-specific based on expression dominance	Constitutive HSP expression consumes ATP; may impair protein turnover	Level D: Wheat HSF families partially characterized

Biological Module	Representative Target or Target Class	Intended Phenotype	Relevant Compound-Stress Scenario	Preferred Editing Strategy	A/B/D Homoeologue Strategy	Principal Trade-Off or Risk	Current Readiness Level
				downregulation			
Senescence regulation	NAC transcription factors (NAM-B1, NAC-S)	Matched senescence timing to stress severity	Combined drought + heat during grain filling	Promoter editing for stress-responsive senescence acceleration or delay	Homoeologue-specific; functional divergence among copies	Stay-green delays remobilization; premature senescence limits grain filling	Level B/C: Natural variation in NAM-B1 affects grain protein; editing not reported
Sugar transport	SWEET and SUT sucrose transporters	Maintained assimilate delivery to grain under stress	Heat + drought during grain filling	Promoter editing for stress-insensitive expression in grain vascular tissue	Homoeologue expression profiling needed	Constitutive overexpression may alter source–sink balance under favourable conditions	Level C/D: Wheat SWEET/SUT expression characterized; editing not reported
Trehalose-6-phosphate signalling	TPS, TPP enzymes	Optimized T6P levels for grain filling under stress	Heat + drought during grain filling	Promoter editing for developmental-stage-specific expression; base editing of catalytic domains	Partial homoeologue editing	Constitutive T6P manipulation disrupts development and flowering time	Level D/E: Rice T6P engineering demonstrated; wheat-specific tools limited
Pollen protection	Pollen-specific heat-shock proteins, sugar transporters	Maintained pollen viability under heat + drought at anthesis	Concurrent heat + drought at anthesis	Pollen-specific promoter engineering; targeted expression of protective proteins	Homoeologue-specific based on pollen expression	Pollen-specific promoters poorly characterized in wheat	Level D/E: Pollen protection mechanisms known; engineering tools undeveloped
Chromatin state regulation	Histone deacetylases (HDACs)	Primed chromatin state at stress-responsive loci	Recurrent stress; stress memory	dCas9–HDAC or dCas9–HAT fusion for locus-specific modification	No homoeologue editing of target loci; effector fusion expressed transgenically	Locus specificity challenging; pleiotropy from off-target chromatin modification	Level E: No wheat epigenome editing reported
Synthetic stress circuits	Engineered stress-responsive promoters + effector genes	Logic-gated stress protection	Multiple specific compound stresses	Targeted insertion of synthetic promoter–effector cassettes	N/A for transgenic cassette insertion	Transgene complexity; regulatory hurdles; stability across environments	Level E: No field-tested synthetic circuits in wheat

Evidence levels are defined in Table S1.

Table S5. Search strategy, screening flow and evidence-classification criteria.

Corresponds to Section 2.1 of the main text. No start date was imposed; the language was restricted to English. The final row records the coverage limitation of the review.

Element	Content
Databases	Web of Science, Scopus, PubMed, Google Scholar
Search date	June 2026
Language / date range	English; no start date imposed
Concept group 1 (species)	wheat / Triticum aestivum / Triticum durum / barley / rice / maize
Concept group 2 (stress context)	compound stress / combined stress / stress combination / sequential stress / recurrent stress / stress memory / priming / acclimation
Concept group 3 (intervention and phenotype)	genome editing / CRISPR / base editing / prime editing / homoeologue / yield stability / yield components / grain filling
Supplementary search	Backward citation tracking from included records
Inclusion criterion (a)	Organism is wheat, or a related cereal able to provide mechanistic support
Inclusion criterion (b)	The temporal structure of the stress treatment is explicitly described (concurrent, sequential, recurrent or single)
Inclusion criterion (c)	At least one phenotype related to yield, a yield component or recovery is reported
Classification rule	All three criteria met → evidence classification (Levels A–E); partially met → contextual citation only, not used as the basis for a claim
Coverage limitation	Evidentiary core 2015–2022; 47 references published in 2023–2025 were added in this revision

Table S6. Operational terms and the minimum experimental design required to support them.

Read together with Table S1 and with the terminology section of the main text (Section 2.2). The 'Evidence state' column records the current status in wheat (W), other cereals (C) or model species (M), following the species-of-origin markers of Table S7.

Term	Operational definition	Minimum experimental design	Evidence state
Acclimation	Adjustment of the response baseline under sustained or repeated stress	Repeated exposure + unexposed control + measurement during the recovery interval	W / Level B–C
Priming	An earlier exposure changes the magnitude or speed of the response to a later exposure	First exposure → recovery interval → re-exposure, with an unprimed control	W / Level C
Somatic memory	A maintained change in response within the lifetime of one plant	Multi-time-point tracking after exposure, until the response difference disappears	W / Level C

Intergenerational effect	Direct influence of parental exposure on the F1 generation, including maternal and seed-provisioning effects	Reciprocal crosses + correction for seed size and provisioning	C–M / Level D–E
Transgenerational inheritance	Effect detectable in unexposed generations (F2 and later)	Assessment in unexposed F2/F3 plants + exclusion of seed and maternal effects	M / Level E (not established in wheat)
Tolerance	Maintenance of function under a given stress	Stress treatment + a defined functional endpoint	—
Resilience	Composite outcome of tolerance, resistance and recovery	Stress + recovery period + whole-season yield	—
Recovery	Rate and extent of functional restoration after stress relief	Time-series measurement during the recovery interval	W / Level C
Yield stability	Low temporal and spatial variation of yield within the target population of environments, with mean yield not significantly below the check	≥3 locations × ≥2 seasons + stability statistics (see Section 8.4)	W / Level A–B

Table S7. Species-of-origin markers and the claim strength each permits.

These markers are applied alongside the evidence hierarchy of Table S1 and are defined in Section 2.3 of the main text. Their purpose is to prevent evidence from non-wheat species from being presented as wheat evidence. The right-hand column lists the specific mis-uses that this revision removes.

**Marker**	**Meaning**	**Permitted claim strength**	**Typical mis-use removed in this revision**
W	Direct evidence from wheat (bread or durum wheat)	Declarative, provided the evidence level (A–E) is stated as well	—
C	Evidence from another cereal (barley, rice, maize)	Hypothetical only: 'suggests', 'may', 'remains to be validated in wheat'; marker must be carried	Root-specific OsNAC10 expression in rice presented as wheat promoter-editing evidence
M	Evidence from a model species such as Arabidopsis	Hypothetical only, with marker carried	Arabidopsis chromatin mechanisms presented as wheat-ready engineering targets
R	Review or meta-analysis	Positioning only; may not be the sole support for a claim	A review used as the only support for a causal claim
(none)	Source of evidence not specified	Not permitted	A Brassica napus homoeologue study cited in

			support of a wheat claim
